# Pneumolysin induced mitochondrial dysfunction leads to release of mitochondrial DNA

**DOI:** 10.1038/s41598-017-18468-7

**Published:** 2018-01-09

**Authors:** Andreas Nerlich, Maren Mieth, Eleftheria Letsiou, Diana Fatykhova, Katja Zscheppang, Aki Imai-Matsushima, Thomas F. Meyer, Lisa Paasch, Timothy J. Mitchell, Mario Tönnies, Torsten T. Bauer, Paul Schneider, Jens Neudecker, Jens C. Rückert, Stephan Eggeling, Maria Schimek, Martin Witzenrath, Norbert Suttorp, Stefan Hippenstiel, Andreas C. Hocke

**Affiliations:** 1 Department of Internal Medicine/Infectious Diseases and Respiratory Medicine, Charité - Universitätsmedizin Berlin, Charitéplatz 1, 10117 Berlin, Germany; 2 Department of Molecular Biology, Max Planck Institute for Infection Biology, Charitéplatz 1, 10117 Berlin, Germany; 3 Institute of Microbiology and Infection, University of Birmingham, Birmingham, B15-2TT UK; 4 Department of Pneumology and Department of Thoracic Surgery, HELIOS Clinic Emil von Behring, Walterhöferstr 11, 14165 Berlin, Germany; 5 Department for General and Thoracic Surgery, DRK Clinics, Drontheimer Strasse 39–40, 13359 Berlin, Germany; 6 Department of General, Visceral, Vascular and Thoracic Surgery, Charité - Universitätsmedizin Berlin, Charitéplatz 1, 10117 Berlin, Germany; 7 Department of Thoracic Surgery, Vivantes Clinics Neukölln, Rudower Straße 48, 12351 Berlin, Germany

## Abstract

*Streptococcus pneumoniae* (*S*.*pn.*) is the most common bacterial pathogen causing community acquired pneumonia. The pore-forming toxin pneumolysin (PLY) is the major virulence factor of *S*.*pn*. and supposed to affect alveolar epithelial cells thereby activating the immune system by liberation of danger-associated molecular patterns (DAMP). To test this hypothesis, we established a novel live-cell imaging based assay to analyse mitochondrial function and associated release of mitochondrial DNA (mtDNA) as DAMP in real-time. We first revealed that bacterially released PLY caused significant changes of the cellular ATP homeostasis and led to morphologic alterations of mitochondria in human alveolar epithelial cells *in vitro* and, by use of spectral live-tissue imaging, in human alveoli. This was accompanied by strong mitochondrial calcium influx and loss of mitochondrial membrane potential resulting in opening of the mitochondrial permeability transition pore and mtDNA release without activation of intrinsic apoptosis. Moreover, our data indicate cellular mtDNA liberation via microvesicles, which may contribute to *S*.*pn*. related pro-inflammatory immune activation in the human alveolar compartment.

## Introduction

Pneumonia remains the most frequent infectious disease and a major cause of morbidity and mortality being responsible for one third of deaths worldwide^1^. The most common pathogen causing community-acquired pneumonia (CAP) is *Streptococcus pneumoniae* (*S*.*pn*.)^2^. We and others already demonstrated that pneumococcal infection of human lung cells and tissue induces a strong pro-inflammatory response and can further lead to cellular death^3–6^. However, it remains to be clarified which microbial factor is the predominant, what cellular events are activated, and if immunological consequences can be expected by the alveolar epithelial damage. The major virulence factor supposed to foster those effects is pneumolysin (PLY)^7^. PLY belongs to the group of cholesterol-dependent cytolysins produced by more than 40 gram-positive bacteria^8^. Autolytic as well as antibiotic-associated bacterial release of PLY allow binding to cholesterol rich cellular membranes leading to toxin oligomerization and subsequent formation of large pores (250–350 Å)^7^. This results in consecutive cellular homeostatic changes, mainly provoked by strong cytosolic calcium ([Ca^2+^]_c_) influx, which may pave the way for cell death induction and an overwhelming immune response leading to acute organ failure^7^ or short- and long-term cardiovascular complications^9^. Such uncontrolled impact on the cellular homeostasis immediately requires well-directed remedies by responsible organelles such as mitochondria, which are known to buffer [Ca^2+^]_c_ excess. Under physiological conditions, well-regulated mitochondrial calcium levels ([Ca^2+^]_m_) contribute to the preservation of membrane potential (ΔΨ_m_) and adequate ATP generation^10^. However, exceeding the mitochondrial capacity can result into loss of those as well as opening of the Ca^2+^-sensitive mitochondrial permeability transition pore (mPTP), organelle swelling, and release of pro-apoptotic factors such as cytochrome C (cytC) or apoptosis inducing factor (AIF)^11,12^. Since the mPTP spans the inner and outer mitochondrial membrane and it has been shown, that its opening contributes to the release of mitochondrial DNA (mtDNA) fragments^13^. Released mtDNA may then serve as intra-, or in case of external release, extracellular DAMP for immune activation^14^. However, the resulting cellular events after [Ca^2+^]_c_ overload are not consequently uniform on the mitochondrion and depend on the biological stimulus. Thus, it remains to be elucidated if PLY induced [Ca^2+^]_c_ influx has the potential to alter [Ca^2+^]_m_ subsequently influencing ΔΨ_m_, ATP generation as well as mPTP associated release of mtDNA.

Clarification of these mechanisms is of pivotal interest for the understanding of *S*.*pn*. induced immune activation since increasing evidence suggests that either cytosolically or micro-environmentally released mtDNA may be recognized by pattern recognition receptors (PRRs) as a DAMP^15^. Thereby, circulating mtDNA may indicate and foster severe inflammation in several disease conditions^14^. For the lung, the alteration of existential mitochondrial functions on the one hand, together with the liberation of mtDNA on the other may dispose a dangerous pathophysiological liaison indicating severe cellular injury^16^ leading to deleterious organ failure^17–19^.

These roles render mitochondria to central organelles for the control of innate immune responses as well as cellular fate in the alveolar compartment and we therefore investigated whether and how PLY affects mitochondrial function in human lung tissue and cultured pulmonary epithelial cells. First, by use of bacterial deletion mutants, we demonstrated that PLY is the major virulence factor altering mitochondrial morphology, motility, ΔΨ_m_, and ATP. Second, the presence of PLY showed massive [Ca^2+^]_m_ influx, opening of the mPTP and, by support of a novel live-cell based assay, mtDNA release independent of mitochondrial caspase activation. Finally, the liberation of mtDNA from the affected cells was found to be concentrated in PLY induced microvesicles.

## Results

### PLY is responsible for alteration of mitochondrial morphology and motility in epithelial cells and human lungs

**Figure 1:**
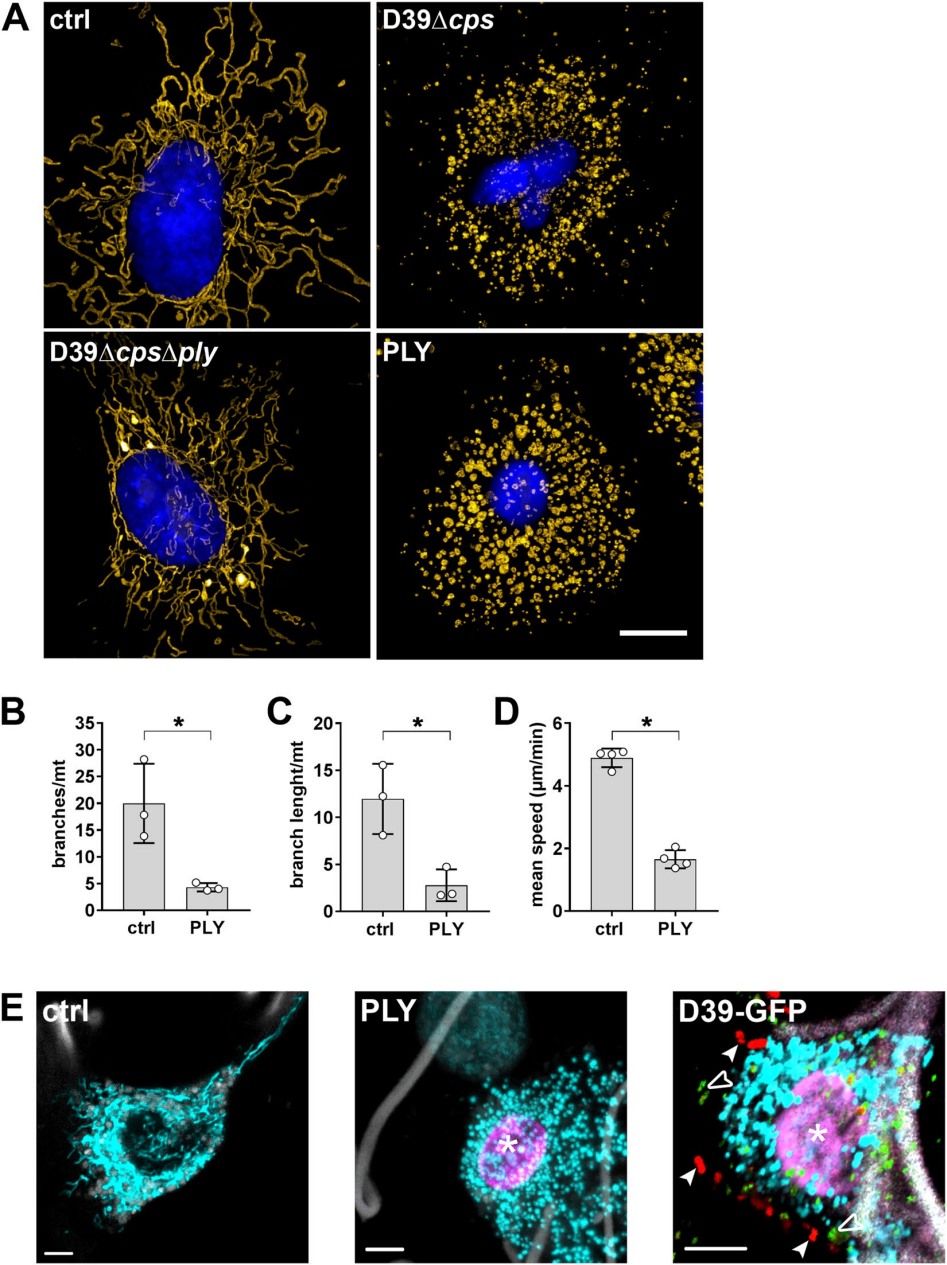
Pneumolysin induces mitochondrial fragmentation and disrupts mitochondrial motility in epithelial cells and human lung tissue. (**A**) A549 cells were labelled with MitoTrackerOrange and subsequently infected with *S*.*pn*. D39Δ*cps* or *S*.*pn*. D39Δ*cps*Δ*ply* for 5 hours, stimulated with 1.0 µg/ml PLY for 15 min or left untreated. Cells were fixed, stained with DAPI and mitochondrial morphology was analysed by structured illumination microscopy. Mitochondria were pseudocoloured using YellowHot LUT and a reconstructed widefield image of the nucleus is shown in blue. Scale bar represents 5 µm. (**B**,**C**) Quantification of mitochondrial morphology in control and PLY treated cells exemplified in (**A**). The mitochondrial network was examined and quantified in stochastically selected mitochondria-rich parts of the cells. Integrative network/shape analysis (number of branches per mitochondrion (**B**) and branch length per mitochondrion (**C**)) was performed (mean ± SD from n = 3 independent experiments, *P < 0.05, one-tailed Mann-Whitney test). (**D**) Quantification of mitochondrial mean velocity in A549 cells immediately before and five minutes after stimulation with 1 µg/ml PLY (mean ± SD from n = 4 independent experiments, *P < 0.05, one-tailed Mann-Whitney test). (**E**) Human lung tissue was labelled with MitoTrackerOrange (cyan) and caspase-3/7 sensor (magenta) and was left untreated (ctrl, left), stimulated with 1.0 µg/ml PLY for 1 h (PLY, middle) or infected with *S*.*pn*. D39-GFP for 12 h (D39-GFP, right). The tissue was analysed by spectral confocal microscopy. Autofluorescence of collagen fibres is shown in grey. In addition GFP-pneumococci are shown in red (arrowheads) and PLY in green (open arrowheads). The asterisk indicates accumulation of caspase-3/7 sensor in the nucleus. Scale bars, 5 µm.

To test whether PLY influences mitochondrial functions, we used the human alveolar epithelial cell line A549 and verified key results in original living human lung tissue explants by a novel spectral live-tissue imaging method, which we established for this purpose. First, analysing mitochondrial morphology in *S*.*pn*. D39Δ*cps*, D39Δ*cps*Δ*ply* (moi 50, 5 h) infected A549 cells or cells exposed to recombinant lytic PLY (1 µg/ml, 15 min) with the super-resolution technique structured illumination microscopy (SIM) showed strong mitochondrial fragmentation and organelle swelling compared to control or infection with PLY deficient mutants (Fig. 1A). To quantify the toxin induced morphological alterations, network analysis of SIM acquired z-stacks in mitochondria rich regions was performed. This analysis showed that PLY induced a 4.6-fold reduction of branches (Fig. 1B) and a 4.4-fold reduced branch length per mitochondrion (Fig. 1C) supporting the conclusion that mitochondrial morphology changes from a typical elongated and filamentous reticular network state to circular organelles. These morphological alterations were accompanied by a significantly reduced mitochondrial motility (mean speed reduction 2.9-fold), which was revealed by automatic tracking of individual mitochondria after confocal live-cell microscopy (Fig. 1D).

Since PLY treatment may cause strong [Ca^2+^]_c_ influx, which may activate cell death mechanisms^20^, we performed live-cell imaging with MitoTracker labelled mitochondria and a caspase-3/7 activity indicator. Cells were infected either with *S*.*pn*. D39Δ*cps* or *S*.*pn*. D39Δ*cps*Δ*ply* and afterwards PLY pores were stained by conventional immunofluorescence with subsequent 3D rendering showing a clear correlation with caspase activation and mitochondrial change of morphology (Supplementary Movies 1 and 2).

Next, we proofed whether these morphological alterations and caspase-3/7 activation were similar in human alveolar epithelial cells embedded in their original three-dimensional tissue architecture. With the same labelling procedure, lungs were exposed to PLY (1 µg/ml, 1 h) or *S*.*pn*. D39-GFP (10^6^ cfu/ml, 12 h) and spectral live-tissue imaging with linear unmixing of auto-fluorescence from overlapping spectra was performed. Untreated tissue showed moving alveolar epithelial cells with elongated, interconnected mitochondria that displayed normal motility (Fig. 1E left, Supplementary Movie 3). In contrast, strong fragmentation of mitochondria as well as caspase-3/7 activation in human lung tissue stimulated with PLY was observed (Fig. 1E middle), which correlated with reduced mitochondrial motility (Supplementary Movie 4). By multi-colour spectral imaging and 3D rendering, the same effect was demonstrated in lungs infected with *S*.*pn*. D39-GFP (arrowheads). Liberated PLY (open arrowheads) from bacteria was additionally visualized by antibody labelling and showed insertion into the cell membranes of the alveolar epithelium in the human lung, which led to mitochondrial fragmentation and caspase-3/7 activation (asterisk, Fig. 1E right, Supplementary Movie 5). These results further underline that *S*.*pn*. induces PLY-dependent changes like mitochondrial fragmentation and disturbs motility, which seems to be an important virulence mechanism in human epithelial cells of the alveolar compartment.

### PLY induced mitochondrial dysfunction and mPTP opening without release of pro-apoptotic factors

**Figure 2:**
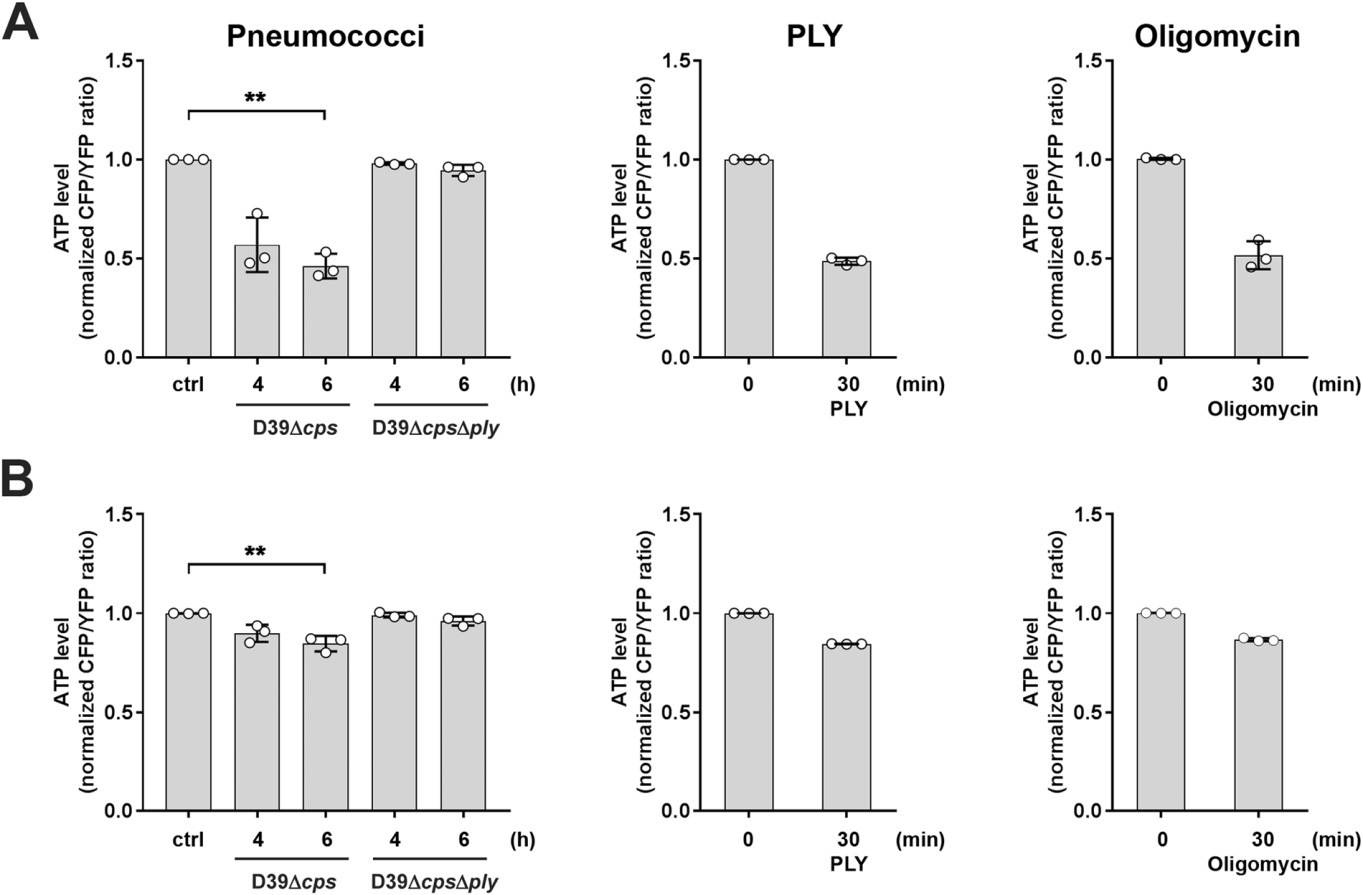
Infection with PLY expressing pneumococci and stimulation with PLY reduces cytosolic and mitochondrial ATP levels. A549 cells were transfected with plasmids encoding FRET-ATP sensors targeted to the cytosol (**A**) and mitochondria (**B**), respectively. 48 hours post transfection, cells were left unstimulated or infected with *S*.*pn*. D39Δ*cps*, *S*.*pn*. D39Δ*cps*Δ*ply* for 4 and 6 h, respectively, or stimulated with 0.25 µg/ml PLY for 30 min. Cells stimulated with 10 µg/ml Oligomycin for 30 min served as positive control. The ATP content is expressed as normalized ratio of the YFP/CFP peak intensity at 530 nm (YFP) and 478 nm (CFP), respectively measured by live spectral-FRET microscopy. Bars represent mean ± SD from n = 3 independent experiments. *P < 0.05, **P < 0.01; Kruskal-Wallis test with Dunn’s post-hoc test.

The morphological alterations of the mitochondria raised the question for critically impaired mitochondrial functions. ΔΨ_m_ is the driving force for ATP production, which was significantly reduced in cells infected with *S*.*pn*. D39Δ*cps* but was not in influenced in cells infected with bacteria deficient for PLY (D39Δ*cps*Δ*ply*) (Supplementary Fig. S1). We next transfected cells with cytosolic or mitochondrial ATP-FRET vectors and used spectral-FRET analysis to assess the effect of either *S*.*pn*. and its PLY deficient mutant or PLY alone on cytosolic and mitochondrial ATP production. A clear dependency of PLY was revealed for reduced ATP in both cellular compartments, which is completely in line with loss of ΔΨ_m_ and distorted morphology (Fig. 2A and B). The comparison to oligomycin exposure (prototypic strong F0 proton channel inhibitor of the ATP synthase serving as positive control) demonstrated that PLY effects on mitochondria were in the same range and must be considered as biologically significant (Oligomycin, Fig. 2A and B).

**Figure 3:**
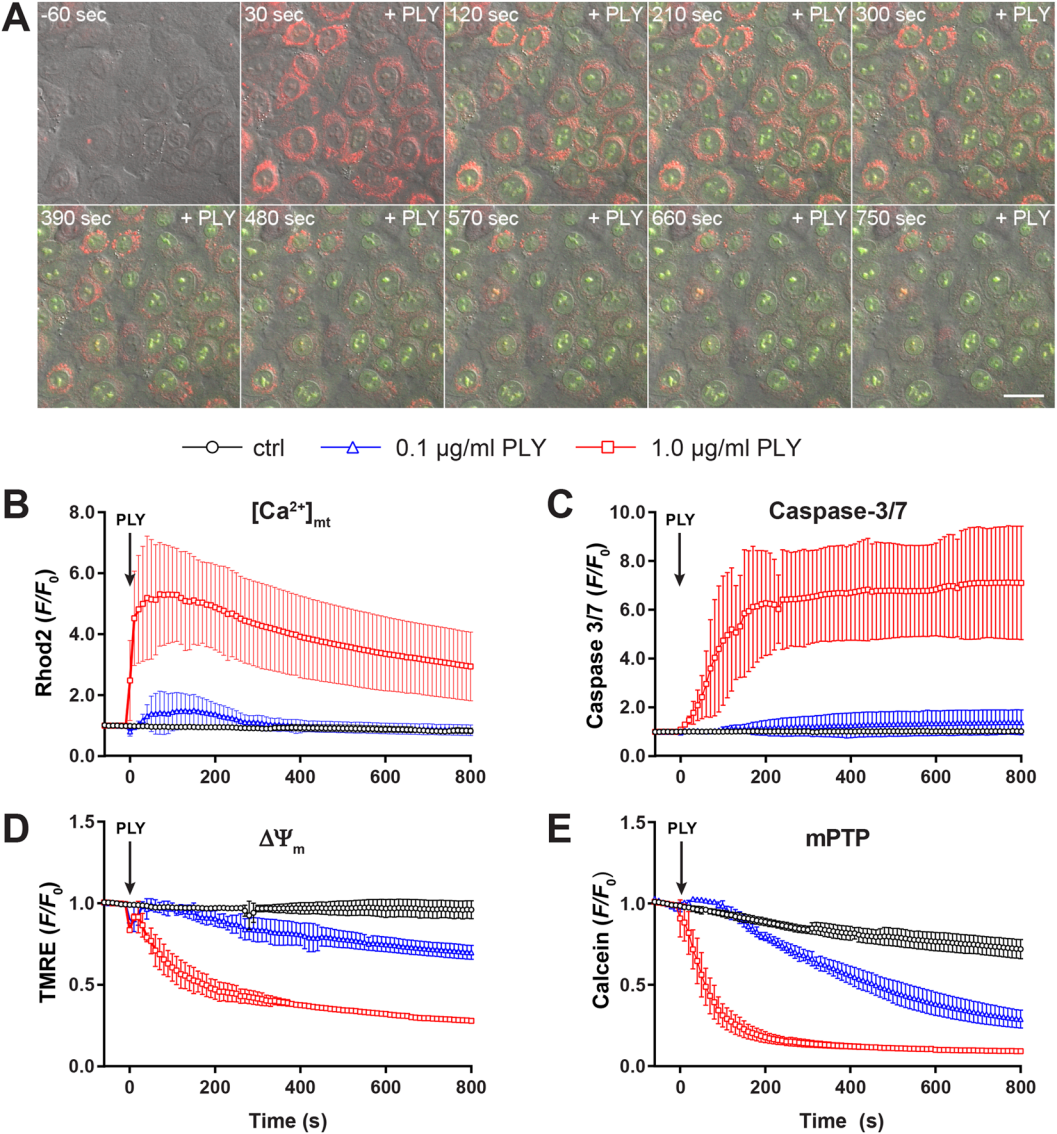
The effect of PLY on mitochondrial calcium, caspase-3/7 activation, membrane potential and opening of mPTP. (**A**) Representative time-lapse images of A549 cells loaded with the mitochondrial calcium sensor Rhod2-AM (red) and stimulated with 1.0 µg/ml PLY in the presence of the caspase-3/7 sensor (green). Scale bar represents 25 µm. (**B**) Quantification of mitochondrial calcium changes (Rhod2 *F*/*F*_0_ ratios) in cells stimulated with 1.0 µg/ml and 0.1 µg/ml PLY or left untreated (mean ± SD from n = 5 independent experiments). (**C**) Quantification of caspase-3/7 induction (*F*/*F*_0_ ratios) in cells stimulated with PLY as described above or left untreated (mean ± SD from n = 3 independent experiments). (**D**) Quantification of changes in mitochondrial membrane potential (TMRE *F*/*F*_0_ ratios) in cells stimulated with PLY as described above or left untreated (mean ± SD from n = 3 independent experiments). (**E**) Opening of the mPTP (*F*/*F*_0_ ratios) in cells stimulated with the indicated amount of PLY or left untreated (mean ± SD from n = 3 independent experiments).

PLY related pore formation is known to increase [Ca^2+^]_c_ concentration^21^, which may play an important role in signal transduction as well as cell death as a result of [Ca^2+^]_m_ overload. Since we already found caspase-3/7 activation accompanied by mitochondrial alteration, we now used live-cell imaging in A549 cells to investigate whether PLY induced changes in [Ca^2+^]_m_ and how this correlates with caspase-3/7 activation. Stimulation with 1 µg/ml PLY revealed an immediate rise in [Ca^2+^]_m_ followed by a slightly lagged induction of caspase-3/7 indicating a correlation of apoptosis induction and mitochondrial dysfunction (Fig. 3A, Supplementary Movie 6). Quantitative analysis showed maximal increase of [Ca^2+^]_m_ approx. 60 s after stimulation with 1 µg/ml PLY (Fig. 3B) that slowly declined whereas the caspase-3/7 signal reached a plateau level after approximately 200 s (Fig. 3C). Treatment with a sub-lytic PLY concentration (0.1 µg/ml) induced a minor rise in [Ca^2+^]_m_ that returned to baseline levels after about 300 s and was accompanied by a comparably low increase in caspase-3/7 activation (Fig. 3B and C, Supplementary Movie 7). Unstimulated cells served as baseline control (Fig. 3B and C, Supplementary Movie 8). Since strong uptake of Ca^2+^ by mitochondria can lead to ΔΨ_m_ depolarization, we correlated loss of ΔΨ_m_ in the same setting and revealed an immediate decrease in cells stimulated with 1 µg/ml PLY (Fig. 3D, Supplementary Movie 9). In contrast, cells treated with 0.1 µg/ml PLY showed slowly decreasing levels of ΔΨ_m_ dissipation over the observation period (Fig. 3D, Supplementary Movie 10), whereas the membrane potential in unstimulated cells remained constant (Fig. 3D, Supplementary Movie 11). The observed [Ca^2+^]_m_ overload has been suggested to induce opening of the mPTP. In order to determine the status of the mPTP we used the Calcein/CoCl_2_ technique. As shown in Fig. 3E, stimulation with 1 µg/ml PLY induced a sharp decrease of the mitochondrial Calcein signal indicative for a rapid opening of the mPTP (Supplementary Movie 12). However, in comparison to the slight effects observed with sub-lytic concentrations of PLY, we found a delayed but significant opening of the mPTP, which was not sufficient to induce caspase-3/7 activation in the measured interval (Fig. 3E, Supplementary Movie 13). The slight decrease in unstimulated cells most likely reflects bleaching/quenching of Calcein without opening of the mPTP (Fig. 3E, Supplementary Movie 14). Taken together, PLY induced dose-dependent rapid [Ca^2+^]_m_ influx, correlated with caspase-3/7 activation and loss of ΔΨ_m_, which was then accompanied by opening of the mPTP. Interestingly, the very low increase of [Ca^2+^]_m_ with sub-lytic PLY was insufficient to induce significant caspase-3/7 activation but finally led to ΔΨ_m_ depolarization and opening of the mPTP indicating a differential regulation of PLY at the mitochondrium.

Therefore, we tested the literature-based hypothesis that opening of the mPTP and the observed strong swelling might be responsible for outer mitochondrial membrane (OMM) rupture for release of pro-apoptotic factors^11,12^. We overexpressed a mitochondrial inner membrane space targeted fluorescent protein (IMS-mEOS) or stained for apoptosis inducing factor (AIF, data not shown) and cytochrome-c (Supplementary Fig. 2A and B). In addition, we partitioned untreated and PLY-treated A549 cells into cytosolic and mitochondrial fractions and analysed the release of cytochrome-c and AIF from mitochondria into the cytosol by western blotting (Supplementary Fig. 2C). Noteworthy, although stimulation with 1 µg/ml PLY caused significant mitochondrial alterations, no release of the addressed proteins from mitochondria was found, indicating mPTP opening is independent from activation of cellular apoptotic pathways on the one hand as well as independent from mitochondrial dysfunction on the other.

### PLY induced mitochondrial DNA release

**Figure 4:**
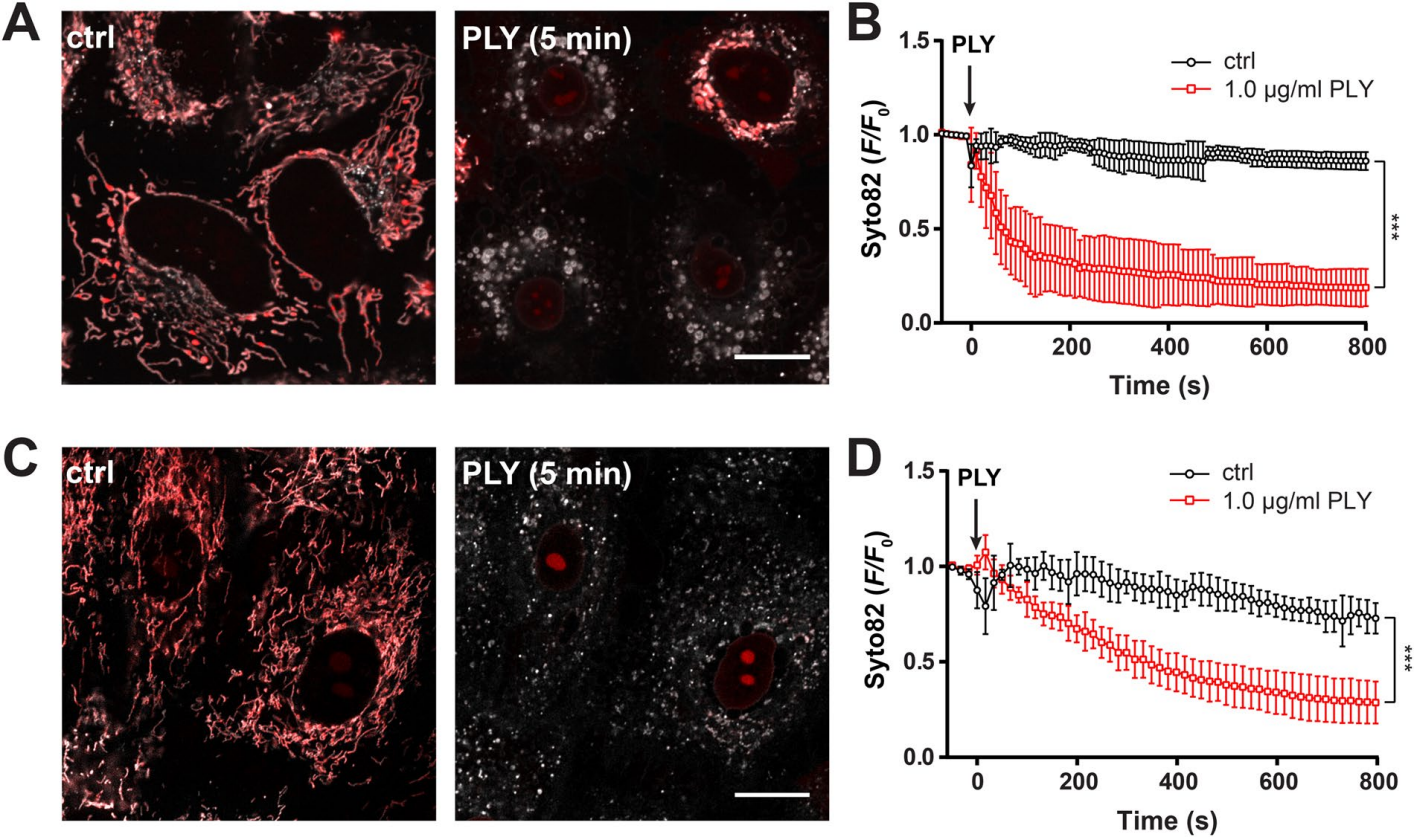
Real-time measurements of PLY induced release of mtDNA in A549 and primary human alveolar epithelial cells. (**A**) Representative confocal 2D plane of mitochondria in A549 cells stained with Syto82 (red) and MitoTrackerOrange (grey) left unstimulated or stimulated with 1.0 µg/ml PLY for 5 min. The scale bar represents 15 µm. (**B**) Quantification of mtDNA release (Syto 82 *F*/*F*_0_ ratios) in cells stimulated as described above (mean ± SD from n = 5 independent experiments, ***P < 0.05, 2-way ANOVA). (**C**) Representative confocal 2D plane of mitochondria in primary human alveolar epithelial cells stained with Syto82 (red) and MitoTrackerDeepRed (grey) left unstimulated or stimulated with 1.0 µg/ml PLY for 5 min. The scale bar represents 15 µm. (**D**) Quantification of mtDNA release (Syto 82 *F*/*F*_0_ ratios) in cells stimulated as described above (mean ± SD from n = 3 independent experiments, ***P < 0.05, 2-way ANOVA).

Mitochondrial dysfunction with opening of the mPTP might lead to release of mtDNA acting as a DAMP^13^. We next used a novel approach described in the Supplementary Note to quantify the release of mtDNA. Therefore, A549 cells were stained with Syto82 and MitoTrackerOrange followed by treatment with 1 µg/ml PLY. Analysis by spectral confocal live-cell microscopy showed a rapid release of Syto82 from mitochondria (Fig. 4A, Supplementary Movies 15 and 16). The kinetics (Fig. 4B) closely resembles the kinetics of mPTP opening (Fig. 3E) and are comparable to those obtained with PicoGreen stained cells (Supplementary Fig. 4A and B). The release of mtDNA from mitochondria was further validated by static immunofluorescence staining of mtDNA in fixed A549 cells treated with 1 µg/ml PLY for different time points (Supplementary Fig. 5). Quantitative analysis showed a significant mitochondrial release of DNA over time, albeit the release kinetics in this assay seem to be slower.

Whether the findings of mtDNA release in A549 cells upon PLY stimulation can be generalized is of importance. Due to technical reasons, Syto82 labelling of mtDNA in living human lung tissue was not possible and we therefore used isolated human pAEC. By using the same approach, we obtained similar results in pAEC with a slightly delayed release kinetic compared to controls (Fig. 4C and D). Taken together, these data show that PLY induces release of mtDNA from mitochondria in human alveolar epithelial cells.

### PLY induced extracellular release of mtDNA

**Figure 5:**
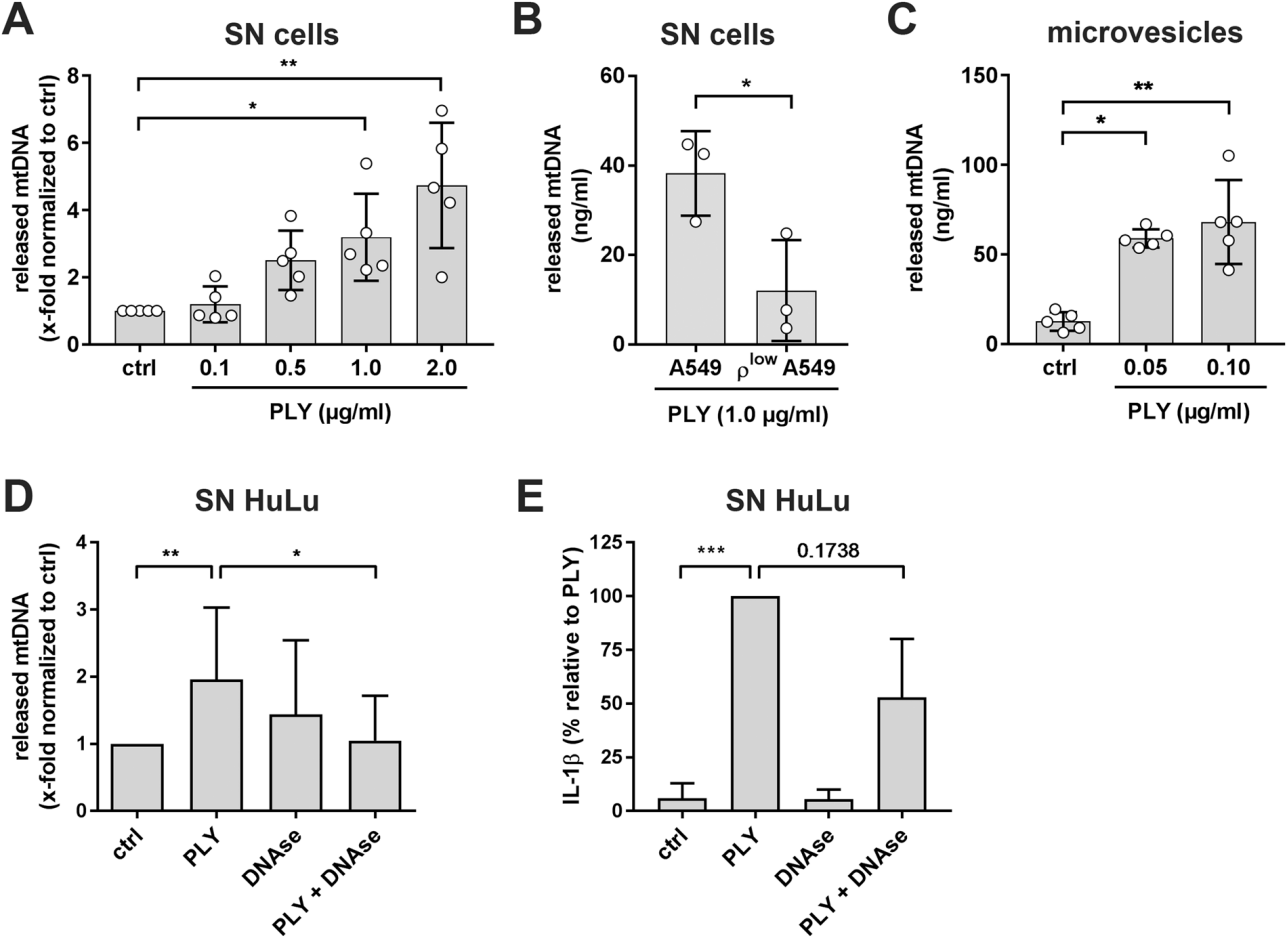
Pneumolysin induces extracellular release of mtDNA. (**A**) Relative quantification of mitochondrial DNA released from A549 cells in the supernatant after stimulation with the indicated amounts of PLY after three hours. Data are expressed as amount of mitochondrial DNA normalized to unstimulated cells (ctrl). Bars represent mean ± SD from n = 5 independent experiments, *P < 0.05, **P < 0.01; Kruskal-Wallis test with Dunn’s post-hoc test). (**B**) Quantification of mitochondrial DNA released from A549 control cells compared to ρ^low^ A549 cells in the supernatant. Cells were stimulated with 1.0 µg/ml PLY for three hours. Bars represent mean ± SD from n = 3 independent experiments, *P < 0.05, one-tailed Mann-Whitney test). (**C**) Quantification of mitochondrial DNA within microvesicles released from A549 stimulated with 0.05 and 0.10 µg/ml PLY for four hours. Bars represent mean ± SD from n = 5 independent experiments, *P < 0.05, Kruskal-Wallis test with Dunn’s post-hoc test. (**D**) Quantification of mitochondrial DNA released from human lung tissue stimulated with 5.0 µg/ml PLY, 5 U/ml DNase or 5.0 µg/ml PLY + 5 U/ml DNase for eight hours. Bars represent mean ± SD from n ≥ 8 independent experiments, *P < 0.05, **P < 0.01; Kruskal-Wallis test with Dunn’s post-hoc test). (**D**) Quantification of IL-1β released from human lung tissue stimulated with 5.0 µg/ml PLY, 5 U/ml DNase or 5.0 µg/ml PLY + 5 U/ml DNase for eight hours. Bars represent mean ± SD from n ≥ 8 independent experiments, ***P < 0.001; Kruskal-Wallis test with Dunn’s post-hoc test).

We next hypothesized that mtDNA fragments may be released from the cytosol into the cellular environment, where they potentially may alert nearby alveolar macrophages (AM) as DAMP for induction of downstream inflammatory responses^19,22^. Moreover, since it has recently been postulated that PLY induced the formation of extracellular vesicles as PLY pores clearance mechanism^23^ we tested if PLY was released freely into the supernatant or packed into small membranous vesicles. First, by qRT-PCR analysis, we compared levels of mtDNA release into the supernatant of PLY-stimulated A549 cells. As shown in Fig. 5A, we observed a dose dependent increase of mtDNA. Due to small amounts and high variation, we had to normalize the data, but most importantly, PLY concentrations leading to a rapid release of mtDNA from the mitochondria also led to significantly 3.2-fold (1.0 µg/ml) and 4.7-fold (2.0 µg/ml) increase of mtDNA into the supernatant (Fig. 5A). This was further validated by the established ρ^low^ A549 cells, which showed a decreased release of mtDNA after 1 µg/ml PLY stimulation (Fig. 5B), comparable to their reduced amount of mtDNA (Supplementary Fig. 3C–E). Next, we treated A549 cells with sub-lytic concentrations of PLY (0.05 and 0.1 µg/ml) to avoid lytic cell damage and to allow regulated cellular processes. From such treated cells, the microvesicle fraction (Supplementary Fig. 6) was isolated and analysed for mtDNA content. Interestingly, compared to supernatants alone, we found a relatively high amount of mtDNA, even with very low doses of PLY indicating a defined alert mechanism of alveolar epithelial cells by DAMP containing microvesicle liberation (Fig. 5C). In addition, we investigated if mtDNA would also be released in living human lung tissue explants. Treatment with 5 µg/ml PLY led likewise to an increased amount of mtDNA whereas treatment with 5 µg/ml PLY in the presence of 5 U/ml DNase significantly reduced mtDNA in supernatants of human lungs (Fig. 5D). Most importantly, treatment with DNase also reduced Il-1β release induced by PLY to a substantial extent (Fig. 5E). Overall, these data suggest that PLY-exposed human lung alveolar epithelial cells release mtDNA from injured mitochondria into the supernatant, most probably via microvesicles, which then might act as DAMP for nearby immune cells.

## Discussion

In the present study, we analysed the effects of pore forming toxin PLY on mitochondria in human alveolar epithelial cells and human lung tissue with regard to release of DAMP molecules. First, we could charge PLY as the main pneumococcal virulence factor responsible for change of mitochondrial morphology, motility, and function. Second, essential processes such as ΔΨ_m_ sustainment and ATP generation were impaired by a strong and rapid [Ca^2+^]_m_ overload, which alone will already have detrimental consequences for the cell. However, the effects on the mitochondria were not uniform and dependent on the exposed toxin dose, which would mirror the *in vivo* situation where cells are confronted with locally variable amounts of bacteria and toxin. This is of particular importance, since PLY induced cell death due to unhampered [Ca^2+^]_c_ influx was concise, especially with high amounts of the toxin. Even these high amounts were not sufficient to release pro-apoptotic factors from the inner membrane space indicating that PLY induced cell death in pulmonary epithelial cells will most probably not follow the intrinsic pathway as assumed elsewhere and discussed below^24–27^. Nevertheless, high toxin amounts and even low sub-lytic PLY were able to reduce ΔΨ_m_ with consecutive opening of the mPTP. This opening of the mPTP was consequently followed by cytosolic release of mtDNA and its subsequent liberation into the microenvironment due to microvesicle constriction.

PLY was shown to induce acute lung failure in various animal models^28,29^ and is of pivotal importance for pneumococcal virulence in the infected host as well as for host-to-host transmission^30^. As part of this, the toxin significantly activated pro-inflammatory pathways (e.g. the inflammasome with IL-1β release) in pulmonary cells and human lung tissue^3,31,32^. Besides, it is well accepted that PLY can activate different cell death pathways such as apoptosis^5,33^, pyroptosis^34^, or necroptosis^35^. However, it remains to be clarified if both, immune activation and cell damage, are meshing mechanisms, which trigger an overwhelming immune response and organ failure in severe cases of CAP. Taken the PLY fostered IL-1β production within the human alveolus, there is currently no evidence if this only results from a direct interaction of the toxin with AM alone or if additional DAMP molecules from injured alveolar epithelial cells are involved in amplifying the signal intensity to detrimental levels^3,36^.

In this regard, mitochondria play a central role since they are responsible for cellular energy supply, contribute to cell death induction, may release mtDNA as DAMP for immune activation, and buffer [Ca^2+^]_c_ overload^37–40^. It seems therefore obvious that PLY induced cell damage, which involves high [Ca^2+^]_c_, could alter such mitochondrial functions as an integral part of pneumococcal pathogenesis, which has also been indicated in neuronal studies^24–27^. These facts motivated us to perform an in-depth analysis of PLY effects on central mitochondrial functions in human lung cells and living human lung tissue.

In many organ tissues, mitochondria constitute highly mobile dynamic structures, in which hitherto only fragmentary understood mechanisms control their size, shape, position and dynamics. Processes like fission, fusion, biogenesis, or mitophagy as well as microtubule-based motility are known to regulate mitochondrial dynamics^41^. In resting lung epithelial cells as well as in human lung tissue, we observed a filamentous presentation of mitochondria, changing to a fragmented, swollen, and rounded phenotype after PLY exposure. Such morphological alterations are supposed to be accompanied by significant changes in mitochondrial functionality, including processes such as calcium regulation, metabolic processes, redox homeostasis, and the regulation of cell fate. Indeed, mitochondrial fragmentation has been shown to limit the spread of Ca^2+^ signals within the mitochondrial network to protect cells from death induction by [Ca^2+^]_m_ overload^42^. In addition, it is well established that strategic localization of mitochondria at subcellular sites of energetic demand and intracellular signaling^43^ as well as distribution and accurate inheritance of mtDNA, is of critical importance to reveal proper cell function over time^41^. Therefore, the observed mitochondrial alterations of morphology and motility were a first indicator for altered mitochondrial functions and induction of cell death by PLY. Interestingly, similar fragmentation processes and blocking of mitochondrial movement was observed in listeriolysin O treated cells^44^. Additionally, the T3SS effector VopE released by *Vibrio cholerae* also modulated mitochondrial dynamics by targeting of Miro GTPases^45^. The observation that enterohemorrhagic *Escherichia coli* hemolysin^46^, *Helicobacter pylori* effector protein VacA^47^, and *Bacillus anthracis* lethal factor^48^ targeted mitochondria, thereby inducing malfunction of mitochondrial pathways critical for cell survival, further highlighted that mitochondria are primary targets for protein toxins released by human pathogenic bacteria.

Well-controlled intracellular [Ca^2+^]_c_ levels are of utmost importance for a balanced cellular signalling, including the regulation of inflammatory pathways and the control of cell death. However, pore formation by PLY is a cellular accident, which has been shown to flood the cell with high [Ca^2+^]_c_ concentrations^21,23^, which might also be true for several other pathogens manipulating host cell physiology by inducing strong Ca^2+^ fluxes^49^. Increased [Ca^2+^]_c_ levels are first buffered by the endoplasmic reticulum and then by mitochondria^39^. Indeed, we observed an immediate and strong increase in [Ca^2+^]_m_ upon PLY stimulation. Massive Ca^2+^ influx into mitochondria can lead to mitochondrial Ca^2+^ overload, which is assumed as a key event in apoptosis induction^20^ but, our data suggest that this response, is not uniform and has to be differentiated for their specific conditions and cell types. Thus, we noted activation of caspase-3/7 accompanying [Ca^2+^]_m_ influx in alveolar epithelial cells as well as in human lung tissue, particularly after higher doses of PLY. However, in contrast to studies performed on neuronal cell types emphasizing a direct PLY interaction with mitochondria and release of AIF as caspase independent cell death pathway^25,26^, we found an early activation of caspase-3/7, which was not accompanied by release of pro-apoptotic factors such as cytochrome-c or AIF from the mitochondrion. Similarly, two other studies on neuronal cells demonstrated a reduction of PLY induced apoptosis by mPTP blockade^24,27^. Again, we likewise found evidence for mPTP opening but no evidence for release of inner membrane space proteins. Since the studies on neuronal cells highlight a direct insertion and pore formation at the mitochondrion, a release of such factors seems reasonable. However, by use of SIM and other advanced imaging techniques, no intracellular PLY was detected, neither *in vitro* nor in *ex vivo* infected human lung explants. These differences to human lung epithelial cells underline that neither [Ca^2+^]_c_ overload by PLY is inevitably resulting in intrinsic apoptosis induction nor that caspase independent apoptotic pathways are pre-dominant after PLY stimulation. It has therefore to be considered that cell type and organ specific factors determine the grade of mitochondrial affection and outcome after a PLY attack.

In line with those studies, we revealed that PLY led to [Ca^2+^]_m_ influx and to dissipation of ΔΨ_m_ accompanied by a decrease of mitochondrial ATP generation and an immediate drop of cytosolic ATP levels. A similar observation was described for listeriolysin O exposed epithelial cells and can therefore undoubtedly be considered as highly critical for the survival of cells affected by pore forming toxins^44^. Moreover, increasing evidence indicated that the activation of the innate and adaptive immune response is a highly energetic, in particular ATP consuming process^50^. The PLY related reduction of the cells capacity to generate ATP may thus significantly reduce the onset and execution of anti-bacterial activities in *S*.*pn*. infected alveoli. However, a direct comparison of human monocyte cell lines (THP-1, U937) with A549 cells showed a higher PLY sensitivity for the epithelial cell type^51^. Thus, innate immune cells such as AM, could be more resilient to PLY and sustain mitochondrial function during the toxin attack compared to alveolar epithelial cells, which probably release DAMP molecules to alert the immune system. Though, the concrete consequences for the human alveolar compartment have to be investigated in further studies.

Mitochondrial Ca^2+^ is the major inducer for opening of the mPTP, which is a large conductance pore spanning both mitochondrial membranes^38^. However, if PLY directly induces mPTP opening was so far not demonstrated. By use of CoCl_2_ based mitochondrial Calcein deposition, we show for the first time PLY dependent opening of the mPTP and its related decrease of ΔΨ_m_ as well as mitochondrial ATP content. In addition to the above-mentioned effects, mPTP opening may allow for the release of mitochondrial factors into the cytosol. In particular, evidence is provided that mtDNA fragments may reach the cytosol via the open pore^13^. Since mitochondrial DNA contains hypomethylated CpG motifs and is therefore immunogenic, its release into the cytosol could be detected by innate immune receptors involving TLR9, NLRP3 and STING as a mitochondrial DAMP molecule, thereby inducing pro-inflammatory and type I interferon responses^22,52,53^.

In order to visualize mtDNA in real-time, we established a novel live-cell imaging assay based on Syto82 (see Supplementary Note). By using this novel live-cell imaging assay, we herein demonstrated that PLY induced the opening of the mPTP with a subsequent release of mtDNA into the cytosol of lung epithelial cells, which may lead to its detection by cytosolic innate immune receptors for induction of pro-inflammatory pathways. However, those local cellular effects might be strongly amplified if mtDNA would be released into the microenvironment or systemic circulation, which would be a reasonable mechanism in PLY induced cellular damage. Several studies provided evidence for correlation of circulating mtDNA with severe disease conditions such as trauma or sepsis and analysis of serum or blood samples from critically ill patients attributed its function not just as a biomarker but as a relevant DAMP^22,54^. Similar effects have been shown in models of acute lung injury and ventilator-associated pneumonia due to *Pseudomonas aeruginosa*^17^. Next to this, intratracheal instillation of mtDNA caused infiltration of immune cells and secretion of pro-inflammatory cytokines such as IL-1β, IL-6, or TNF-α via the activation of p38 MAPK pathways^18^, which we have demonstrated to be likewise up-regulated in *S*.*pn*. infection of human lung tissue^6^. Therefore, we tested if cytosolic mtDNA would be secreted into the supernatant after PLY and found evidence in human alveolar epithelial cells and even human lung tissue and we are able to link mtDNA release to immune activation in human lung tissue as illustrated by a reduced release of IL-1β into the supernatant of tissue treated with PLY in the presence of DNase. However, measurement of variable amounts of mtDNA from the unfractionated supernatants led us wonder if the DAMP might be protected by and packed in microvesicles. Indeed, Guescini *et al*. has demonstrated that exosomes may contain mtDNA and a couple of mitochondrial proteins^55^. Microvesicles are a heterogeneous group of tiny exosomes secreted by both, eu- and prokaryotes under several conditions but have been shown to effectively contribute to host-pathogen interactions and activation of immune pathways^56^. Isolation and analysis of microvesicles from supernatants of PLY stimulated human alveolar epithelial cells clearly revealed stable and significant amounts of mtDNA. Therefore, it is tempting to speculate that PLY related release of mtDNA containing microvesicles in the human alveolus can impact by several ways on the innate immune response. One obvious would be their activation of innate immune receptors in AM, where PLY induced inflammasome activation and other pro-inflammatory mediators may be further fostered. Ongoing studies have to elucidate the role for alveolar released mtDNA in the human alveolus under infectious conditions.

Taken together, our results provide evidence, that pneumococcal PLY disturbs mitochondrial functions in human lungs by Ca^2+^ overload, leading to breakdown of ΔΨ_m_ and opening of the mPTP. By using newly established multi-colour spectral live-tissue imaging and a new live-cell assay, we could follow mitochondrial damage in the living human alveolar compartment and found mtDNA release in PLY treated human alveolar epithelial cells. mtDNA is released independent of activation of (intrinsic) apoptotic pathways, but, by a regulated liberation in microvesicles, which may act as a pro-inflammatory DAMP indicating severe lung tissue damage to the host. Bacterial-toxin related disturbance of vital mitochondrial function should be considered as a significant cause of tissue damage in severe infections and therapeutic PLY inhibition thus promise to reduce significantly pneumococcal tissue damage and organ dysfunction by protecting mitochondrial function and reducing immune activation.

## Methods

### Ethics Statement

Fresh human lung explants were obtained from adult patients undergoing lung resection at thoracic surgery centres. Written informed consent was obtained from all patients. The study was approved by the local institutional review board (Ethics committee of the Charité - Universitätsmedizin Berlin, Germany, EA2/079/13) and performed in accordance with the approved guidelines.

### Materials

MitoTracker^®^ Green FM, Orange CMTMRos, and Deep Red FM, Image-IT^®^ LIVE Mitochondrial Transition Pore Assay Kit, SYTO^®^ 82, tetramethylrhodamine ethyl ester perchlorate (TMRE), CellEvent™ Caspase-3/7 Green Detection Reagent, JC-1 and secondary Alexa Fluor conjugated antibodies were obtained from Life Technologies (Darmstadt, Germany). The antibody against DNA was purchased from Progen Biotechnik (Heidelberg, Germany), the anti-Tom20 antibody (sc-11415) was obtained from St. Cruz Biotech (Heidelberg, Germany) and anti-cytochrome-c antibody was obtained from BD (#556432, Heidelberg, Germany). Pneumolysin (PLY) was produced and purified as previously described^57^. 16% formaldehyde without methanol was obtained from electron microscopy science (Hatfield, PA) and CitiFluor™ CFM3 mounting medium was from Citifour (London, UK). Plasmids encoding the AT1.03^YEMK^ fluorescence resonance energy transfer (FRET) sensors targeted to the cytosol (cytoATeam) and mitochondria (mitoATeam), respectively were kind gifts from Hiromi Imamura (Kyoto University)^58^. pBabe-puro-IMS-RP was a gift from Peter Sorger (Addgene plasmid #24535) and mEos3.2-N1 was a gift from Michael Davidson (Addgene plasmid #54525).

### Bacterial strains

The *S*. *pneumoniae* strains used in this study are the D39 wildtype strain, a D39 derived capsule locus (*cps*) deletion mutant D39Δ*cps*, the *cps*/pneumolysin double mutant D39Δ*cps*Δ*ply*, and a GFP-expressing D39 strain (D39::GFP). *S*.*pn* strains were cultivated in Todd-Hewitt broth (BD, Heidelberg, Germany) supplemented with 0.5% yeast extract (BD, Heidelberg, Germany) to mid-log phase (*A*_600_ = 0.35–0.40) at 37 °C and 5% CO_2_ or grown on Columbia blood agar plates (BD, Heidelberg, Germany).

### Cell culture and lung explant tissue

The human lung alveolar epithelial cell line A549 (ATCC, CCL-185) was cultured in Ham’s F12 medium (Biochrome, Berlin, Germany) supplemented with 10% (v/v) fetal bovine serum (FBS; Capricorn Scientific GmbH, Ebsdorfergrund, Germany) and 2 mM L-glutamine (Invitrogen Life Technologies) at 37 °C and 5% CO_2_. A549 cells were infected with *S*.*pn.* strains using a multiplicity of infection (MOI) of 50 in Ham’s F12 medium supplemented with 2% FBS for the indicated periods at 37 °C/5% CO_2_.

Primary human type II alveolar epithelial cells (AEC-II) were isolated from human lung tissue as described previously^36^. Briefly, human lung tissue was finely minced and transferred into HBSS. The tissue was filtered through a sterile gauze 70 µm strainer and digested with trypsin type I (SERVA, Amstetten, Austria) and elastase (Merck, Darmstadt, Germany) for 40 min at 37 °C. After inactivation of enzymes and recovery of the cells using 30% FBS (GE Healthcare LifeSciences, Freiburg, Germany), the digested tissue was filtered through a sterile gauze 100 µm strainer. Cells were seeded and expanded on irradiated NIH-3T3 (German Collection of Microorganisms and Cell Cultures (DSMZ), ACC 59) feeder cells in F-Medium. For stimulation experiments AEC-II were harvested by trypsinisation, separated from feeder cells and seeded in ibidi slides in DMEM containing 10% (v/v) FBS and 2 mM L-glutamine.

ρ^low^ A549 cells were generated by incubation of A549 cells with low doses of ethidium bromide in DMEM (high glucose, supplemented with 10% FBS, glutamine, pyruvate and 50 µg/ml uridine) for six to seven passages (approx. 2 weeks).

Tumor-free normal lung tissue was processed and inoculated with 1 × 10^6^ cfu/ml of *S*.*pn*. D39-GFP for 12 h, as described previously^3^.

To label mitochondria, cells were incubated with 100 nM of the respective MitoTracker^®^ dyes dissolved in dimethyl sulfoxide, for 30 min at 37 °C/5% CO_2_. Membrane potential was determined by loading cells with 7.7 µM JC-1 or 10 nM tetramethyl-rhodamine methyl ester (TMRE) for 30 min at 37 °C/5% CO_2_ and washed three times with HBSS. TMRE is used in the nonquenching mode to assess ΔΨ_m_, and therefore a reduction in TMRE fluorescence represents mitochondrial depolarization. To visualize mPTP opening by the Calcein-CoCl_2_ assay mitochondria were stained as described above and subsequently loaded with 1 mM Calcein acetoxymethyl ester and CoCl_2_ as described by the manufacturer. Mitochondrial calcium influx was determined by loading the cells with 4.45 µM Rhod2-AM for 30 min using FBS-free medium followed by three washing steps with HBSS.

### Intracellular ATP measurement

Mitochondrial ATP levels were measured by transfecting A549 cells seeded in 8-well µ-slides (ibidi, Munich, Germany) with 0.4 µg plasmids encoding cytoATeam or mitoATeam using ViaFect transfection reagent (Promega, Wisconsin, USA). 48 hours post transfection, cells were left unstimulated, infected with pneumococci as described above or stimulated with 0.25 µg/ml PLY. Stimulation with 10 µg/ml oligomycin A served as positive control. ATP concentration was determined by spectral confocal microscopy using a LSM 780 confocal laser-scanning microscope equipped with a 40×/1.2 NA C-Apochromat driven by Zen 2012 software (Carl Zeiss, Jena, Germany). Lambda stacks were acquired using 458 nm excitation and the ATP content is expressed as normalized ratio of the YFP/CFP peak intensity at 530 nm (YFP) and 478 nm (CFP), respectively.

### Indirect immunofluorescence and microscopy

Cells grown in ibibi slides were stained with 100 nM MitoTracker Orange, stimulated as indicated, fixed with cold methanol at −20 °C for 20 min or 3.7% formaldehyde in PBS for 15 min at room temperature and permeabilized with 0.5% Triton-X100 in PBS. Cells were blocked with PBS containing 5% goat serum, 1% bovine serum albumin (BSA) and 0.2% Tween-20. After washing with PBS, cells were incubated with primary antibodies diluted in PBS/1% BSA/0.2% Tween-20 over night at 4 °C. After three washes with PBS, cells were subjected to an overnight incubation with AlexaFluor 488/555-conjugated secondary antibodies at 4 °C. Cells were washed three times with PBS and nuclei were stained with DAPI for 20 min and subsequently washed three times with PBS. Cells were mounted in CFM3 mounting medium or examined directly in PBS.

Confocal microscopy was performed on a LSM 780 confocal laser-scanning microscope driven by Zen 2012 software. For live cell imaging experiments cells were imaged at 37 °C/5% CO_2_ in phenol red-free medium using a 40×/1.2 NA C-Apochromat or a 63×/1.4 NA Plan-Apochromat objective (mitochondrial motility). 3D high resolution image stacks with a z-step size of 0.25 or 0.30 μm per plane were acquired using a 63×/1.4 NA Plan-Apochromat objective and the pinhole set to 1 airy unit in sequential imaging mode to avoid bleed-through of fluorescence emission.

For Airyscanning microscopy an LSM 880 with Airyscan detector equipped with a 63×/1.4 NA Plan-Apochromat objective controlled by Zen 2 software was used. Cells were maintained at 37 °C/5% CO_2_ for time-lapse imaging.

Structured illumination microscopy (SIM) was performed on an ELYRA PS.1 microscope with a 63×/1.40 Plan-Apochromat objective with 405, 488, and 561 nm excitation lines (Carl-Zeiss, Jena, Germany) as described previously^59^. In brief, SIM images were acquired with five phase shifts and three or five rotations of the structured illumination grid and a z-step size of 120 nm. Resulting stacks were processed in ZEN 2.1 software using automatically determined parameters.

Detailed information on imaging processing and analysis can be found in the SI methods.

### Isolation of mitochondrial DNA and qRT-PCR

Isolation of extracellular mtDNA from A549 and human lung tissue stimulated with the indicated amounts of PLY was done after 3 and 8 hours, respectively. The supernatant containing the mtDNA was collected and centrifuged at 2100 × *g* for 10 min to remove cell debris. For microvesicle isolation, A549 were treated with PLY (50 or 100 ng/ml) in serum-free media for 4 hours. The microvesicles were isolated from the conditioned media as previously described with minor modifications^60^. Microvesicles were washed with PBS and processed for mtDNA analysis. Mitochondria were isolated using the Mitochondria Isolation Kit for Cultured Cells (Thermo Fisher, Darmstadt, Germany) as described by the manufacturer. mtDNA was isolated using the QIAamp DNA Mini Kit (Qiagen, Hilden, Germany). Quantitative PCR was performed using TaqMan assays (MT-CYB, Hs 02596867_s1, Life Technologies) on an ABI 7300 instrument. The amount of mtDNA in the supernatant was calculated based on a standard curve generated using purified mtDNA from isolated mitochondria. mtDNA copy number analysis was performed usingTaqMan assay for mtDNA and nuclear DNA (GAPDH Hs 99999905_m1, Life Technologies). Relative mtDNA copy number was calculated as the ratio of mtDNA abundance versus nuclear DNA abundance and normalized to the control group.

### Statistical analysis

GraphPad Prism 7 (Version 7.01) software was used for the statistical analysis. Values are expressed as means ± SD from at least three independent experiments. The Mann-Whitney test was used for comparison of two groups and the one-way/two-way ANOVA Kruskal-Wallis with Dunn’s post-hoc test was used for comparison of three or more groups.

### Data Availability

All data generated or analysed during this study are included in this published article and its Supplementary Information files.

## Electronic supplementary material

Supplementary Information

Supplementary Movie 1

Supplementary Movie 2

Supplementary Movie 3

Supplementary Movie 4

Supplementary Movie 5

Supplementary Movie 6

Supplementary Movie 7

Supplementary Movie 8

Supplementary Movie 9

Supplementary Movie 10

Supplementary Movie 11

Supplementary Movie 12

Supplementary Movie 13

Supplementary Movie 14

Supplementary Movie 15

Supplementary Movie 16
