# Supplementary material for: Pneumolysin induced mitochondrial dysfunction leads to release of mitochondrial DNA
Source: Sci Rep. 2018 Jan 9;8:182. doi: 10.1038/s41598-017-18468-7 (PMC5760655; doi:10.1038/s41598-017-18468-7)
Supplement: Supplementary file 1 — Supplementary Information [file 41598_2017_18468_MOESM1_ESM.pdf]

Supplementary Information

Pneumolysin induced mitochondrial dysfunction leads to release of mitochondrial DNA

Andreas Nerlich[1], Maren Mieth[1], Eleftheria Letsiou[1], Diana Fatykhova[1], Katja Zscheppang[1], Aki Imai-Matsushima[2], Thomas F. Meyer[2], Lisa Paasch[1], Timothy J. Mitchell[3], Mario Tönnies[4], Torsten T. Bauer[4], Paul Schneider[5], Jens Neudecker[6], Jens C. Rückert[6], Stephan Eggeling[7], Maria Schimek[7], Martin Witzenrath[1], Norbert Suttorp[1], Stefan Hippenstiel[1], Andreas C. Hocke[1*]

[1]Department of Internal Medicine/Infectious Diseases and Respiratory Medicine, Charité - Universitätsmedizin Berlin, Charitéplatz 1, 10117 Berlin, Germany.

[2]Department of Molecular Biology, Max Planck Institute for Infection Biology, Charitéplatz 1, 10117 Berlin, Germany

[3]Institute of Microbiology and Infection, University of Birmingham, Birmingham, B15-2TT, UK

[4]HELIOS Clinic Emil von Behring, Department of Pneumology and Department of Thoracic Surgery, Chest Hospital Heckeshorn, Walterhöferstr. 11, 14165 Berlin, Germany.

[5]Department for General and Thoracic Surgery, DRK Clinics, Drontheimer Strasse 39-40, 13359 Berlin, Germany.

[6]Department of General, Visceral, Vascular and Thoracic Surgery, Charité - Universitätsmedizin Berlin, Charitéplatz 1, 10117 Berlin, Germany.

[7]Department of Thoracic Surgery, Vivantes Clinics Neukölln, Rudower Straße 48 12351, Berlin, Germany.

*Address for correspondence

Andreas C. Hocke (MD), Charité - Universitätsmedizin Berlin, Department of Internal Medicine/Infectious Diseases and Respiratory Medicine, Charitéplatz 1, 10117 Berlin, Germany, Tel: +49 30 450 553477, e-mail: andreas.hocke@charite.de

Supplementary Methods

IMS-mEOS plasmid construction

mEOS targeted to the mitochondrial inner membrane space (IMS) was generated by amplifying the region coding for the IMS sequence (comprising bp 1-147 of Smac-1) from the plasmid pBabe-puro-IMS-RP (Addgene plasmid #24535) using the primers 5'-GCATACGCTAGCACCATGGCGGCTCTGAAGAGTTG-3' and 5'-CTACGTACCGGTCCCGGATCTCCTGATCCTCCTG-3'. The resulting PCR fragment was cloned into mEOS3.2-N1 (Addgene plasmid #54525) via *Nhe*I/*Age*I restriction sites. The construct was sequenced before use and purified with the Endofree® plasmid maxi kit (Qiagen, Hilden, Germany).

Image processing and analysis

For quantification of $[Ca^{2+}]_m$ influx, changes in $\Delta\Psi_m$, caspase-3/7 activation and opening of mPTP in A549 cells a rectangular ROI was placed in the image sequence acquired as 2 × 2 tile scan containing approx. 250 cells and the intensity of all pixels within the ROI was averaged for each frame. Subsequently the F_i/F_0 ratio was calculated, where F_i is the fluorescence signal at frame i and F_0 is the baseline fluorescence signal averaged over a 60 s period at the beginning of the recording.

For quantification of Syto82 in combination with MitoTracker Green, fluorescence time-lapse series of stained cells acquired in lambda mode as 2 × 2 tile scan containing approx. 250 cells (A549) or 50 cells (hAECs) were first spectrally unmixed in Zen 2012. Next, the MitoTracker channel was spatially processed using a Gaussian filter with a radius of 1, followed by applying a linear contrast stretch and binarization using Otsu method in Fiji/ImageJ. Using this mask, the intensity in the Syto-82 channel was measured in the mitochondrial area.

If indicated, images were deconvolved using Huygens® Essential 15.10 (Scientific Volume Imaging, Hilversum, NL) and 3D-stacks are displayed as maximum intensity projections (MIPs) and adjusted for brightness and contrast in Zen or ImageJ/Fiji.

Quantification of mitochondrial morphology and motility

Quantification of mitochondrial morphology was done as described previously[1]. In brief, 4-5 randomly chosen field of views containing 3-5 cells were imaged resulting in 14-18 analysed cells per independent experiment. Maximum intensity projections of SIM 3D stacks in stochastically selected mitochondria-rich parts of the cells were skeletonized following thresholding using the Huang method and using the "Skeletonize 2D/3D" plugin in Fiji/ImageJ. The resulting vectorised mitochondrial skeleton was used to identify and count/measure branches, branch points and end points.

For quantification of mitochondrial motility, images were collected directly before and 5 min after stimulation with PLY in one randomly chosen field of view containing 14-29 cells per independent experiment. Images were acquired every 3.13 seconds over a total imaging time of 2.04 min at a resolution of 0.132 µm/pixel (1024 × 1024 pixels). Mitochondrial motility was analysed with Imaris (v8.1.2, Bitplane AG, Zürich, Switzerland). Mitochondrial trajectories were generated using the following settings: estimated diameter = 1.32 µm, background subtraction, maximum tracked distance = 1.5 µm, maximum gap size= 2 frames, tracking algorithm: autoregressive motion. Tracks with a duration less than 0.5 min were excluded.

Quantification of anti-DNA staining in mitochondria

Quantification of anti-DNA staining was performed using Imaris software (v8.1.2, Bitplane AG, Zürich, Switzerland) in 3 randomly chosen areas containing approx. 20

cells per experiment. First, a mask was generated based on the DAPI signal and applied to the anti-DNA channel to exclude the nuclear DNA signal. Next, the mitochondrial volume was calculated based on the MitoTracker signal using an appropriate intensity thresholding. All volumes smaller than 0.1 μm^3 were excluded. Finally, the fluorescence intensity within the mitochondrial volume in the masked anti-DNA channel was quantified.

Subcellular fractionation and western blotting

Cytosolic and mitochondrial fractions were generated using the Qproteome Mitochondrial Isolation Kit (Qiagen, Hilden, Germany) according the manufacturer's recommendation. Equal protein amounts were resolved on 13% SDS-PAGE gels and blotted onto PVDF membranes. Membranes were blocked in 5% skimmed milk for 1 hr hour at room temperature, washed and incubated overnight at 4°C with the following primary antibodies: anti-AIF (D-20, St. Cruz, 1:1000), anti-Cytochrome-C (clone7h8.2C12, Biolegend 1:1000), anti-GAPDH (D16H11, Cell Signalling, 1:5000), and anti-Tom20 (FL-145, St. Cruz, 1:1000). After washing, the blots were incubated for 1 hour with secondary antibodies against goat, rabbit or mouse IgG (St. Cruz, Heidelberg, Germany) and washed. All washing steps were performed in PBS/0.05% Tween® 20 (3 × 5 min). Blots were developed using Pierce ECL Western Blotting Substrate (Thermo Fisher, Bonn, Germany) on Carestream Kodak BioMax light films (Sigma, Taufkirchen, Germany)

Supplementary Note: Visualization of mtDNA in living alveolar cells by Syto82

Opening of the mitochondrial permeability transition pore (mPTP) is associated with potential release of mtDNA fragments[2], which may promote immune activation. In order to visualize mtDNA in real-time, we established a novel live-cell imaging assay based on Syto82, a nucleic acid dye belonging to the dye Syto® family.

It has been demonstrated that some members of the Syto® dye family of fluorescent nucleic acid dyes can incorporate into mitochondria[3] and we found that Syto82 specifically labels mtDNA in A549 cells. Using high-resolution confocal microscopy (Airyscan®), of living A549 cells stained with Syto82 and MitoTracker-DeepRed, we observed a distinct punctate staining pattern distributed throughout the mitochondrial network (Supplementary Fig. 3A). Most importantly, the size, shape, and distribution of the detected Syto82 staining in living cells resembled that of mitochondrial nucleoids detected in living cells stained with PicoGreen (Supplementary Fig. 4C) and in fixed cells stained with an anti-DNA antibody (Supplementary Fig. 3B). This mtDNA pattern is completely in line with the localization found in a previous study[4]. To further prove the specificity of Syto82 staining, we depleted mtDNA in A549 cells by treatment with ethidium bromide resulting in the generation of ρ^{low} A549 cells[5]. Staining of living ρ^{low} A549 cells with Syto82 resulted in a reduced number of puncta as compared to non-depleted A549 cells and a significantly reduced mean fluorescence intensity of 60.85% (Supplementary Fig. 3C). A similar 61.24% reduction of mitochondrial DNA content was observed when fixed ρ^{low} A549 cells were stained with the anti-DNA antibody and the mean mitochondrial fluorescence was compared to non-depleted A549 cells (Supplementary Fig. 3D). Therefore, Syto82 staining is a sensitive and accurate new staining method for quantifying mtDNA in live-cell imaging.

The ability of Syto82 to stain mtDNA may relate to its cationic charge, which may allow preferential accumulation of the dye within mitochondria in a similar manner to ethidium bromide[6]. To further validate Syto-82 staining we used PicoGreen. Staining of mtDNA by this dye has been suggested to be unaffected by the mitochondrial membrane potential once the dye is in the mitochondria[7]. Kinetic analysis of cells stained with PicoGreen and treated with 1.0 µg/ml PLY revealed similar kinetics (Supplementary Fig. 4A and B) as observed with Syto-82 stained cells. We found that Syto82 can be easily used to stain mtDNA in living cells. Its specificity was confirmed by co-localization with mitochondrial markers and the absence of staining in ρ^{low} A549 cells. Syto82 visualization of mtDNA distribution is consistent with previous studies, utilizing a variety of methods[7-10] indicating that mtDNA in alveolar epithelial cells is organized into punctuate nucleoid structures within mitochondria. Like ethidium bromide[11], Syto82 primarily stains mitochondrial DNA in healthy alveolar cells but not nuclear DNA as shown recently for PicoGreen[7]. However, in contrast to PicoGreen, nucleoid staining cannot be fixed with formaldehyde or methanol, thus it does not allow subsequent visualization of other cellular components with antibodies. Overall, staining of mtDNA by Syto82 now allow it's tracking by real-time microscopy, which will indeed be helpful for many future studies in the field.

Supplementary Figures

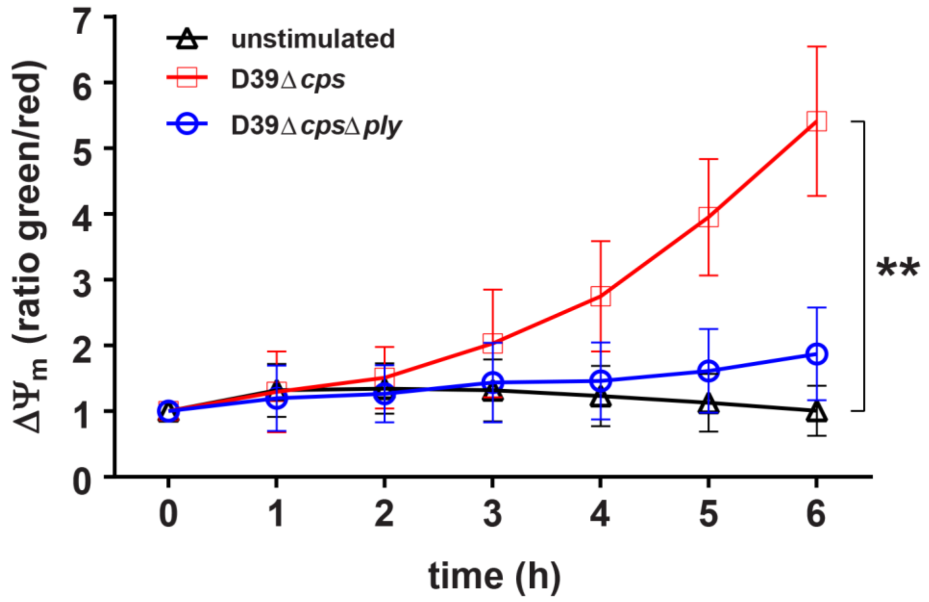

Supplementary Figure 1. *S.pn.* infection of A549 cells reduces mitochondrial membrane potential in a pneumolysin dependent manner. A549 cells were loaded with JC-1 and infected with *S.pn.* D39Δ*cps* or *S.pn.* D39Δ*cps*Δ*ply* (moi 50) for 6 h. Data are expressed as normalized ratio of red/green fluorescence measured in a plate reader at 37°C. An increase of this ratio indicates a reduction of $\Delta\Psi_m$. Data represent mean ± SD from n = 4 independent experiments, *$P < 0.05$, Kruskal-Wallis test with Dunn's post-hoc test).

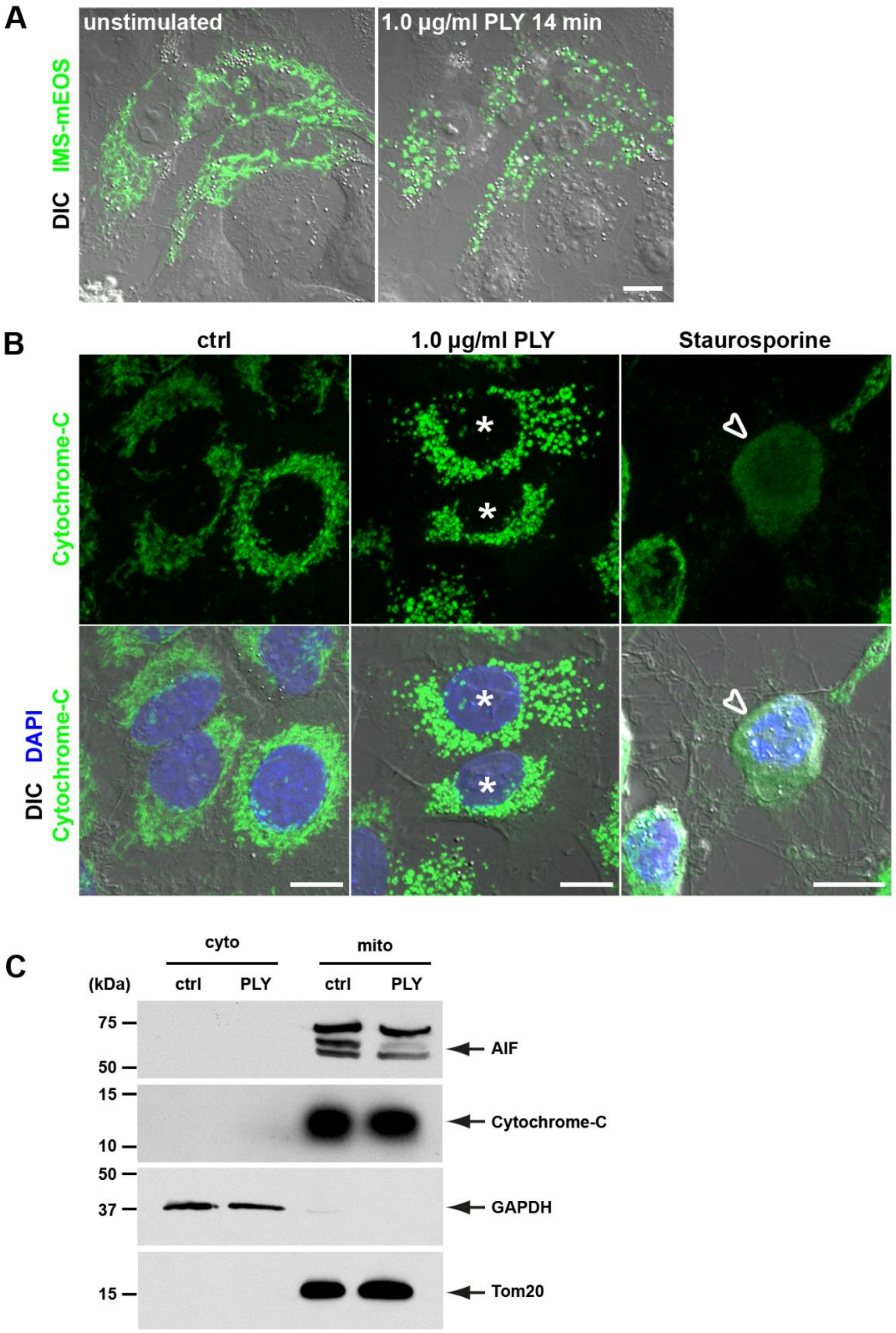

Supplementary Figure 2. PLY stimulation does not lead to release of pro-apoptotic factors from mitochondria. (A) A549 cells were transfected with a mEOS variant targeted to the inner membrane space of mitochondria (IMS-mEOS; for plasmid construction see SI methods 1). 48 hours post transfection, cells were stimulated with 1.0 µg/ml PLY for 14 min and analysed by confocal time-lapse microscopy. IMS-mEOS

is shown in green and cell morphology was visualised by differential interference contrast (DIC). Scale bar represents 10 µm. (B) A549 cells were left unstimulated (ctrl) or stimulated with 1.0 µg/ml PLY for 15 min. As a positive control, cells were stimulated with 4 µM staurosporine for 16 hours. Cells were fixed and immunostained for cytochrome-c and analysed by confocal microscopy. DNA was stained with DAPI (blue) and cell morphology was visualised by DIC. Representative maximum intensity projections are shown. Asterisks indicate cells with fragmented mitochondria and still mitochondrially localized cytochrome-c. Open arrowheads indicate a cell with released cytochrome-c from mitochondria into the cytoplasm. Scale bars represents 10 µm. (C) A549 cells were stimulated with 1 µg/ml PLY for 15 min or left untreated and subjected to subcellular fractionation. Cytosolic (cyto) and mitochondrial (mito) fractions were analysed by western blot using the indicated antibodies. The relative molecular weights (kDa) of a prestained protein ladder are indicated on the left. Data are representative of three independent experiments.

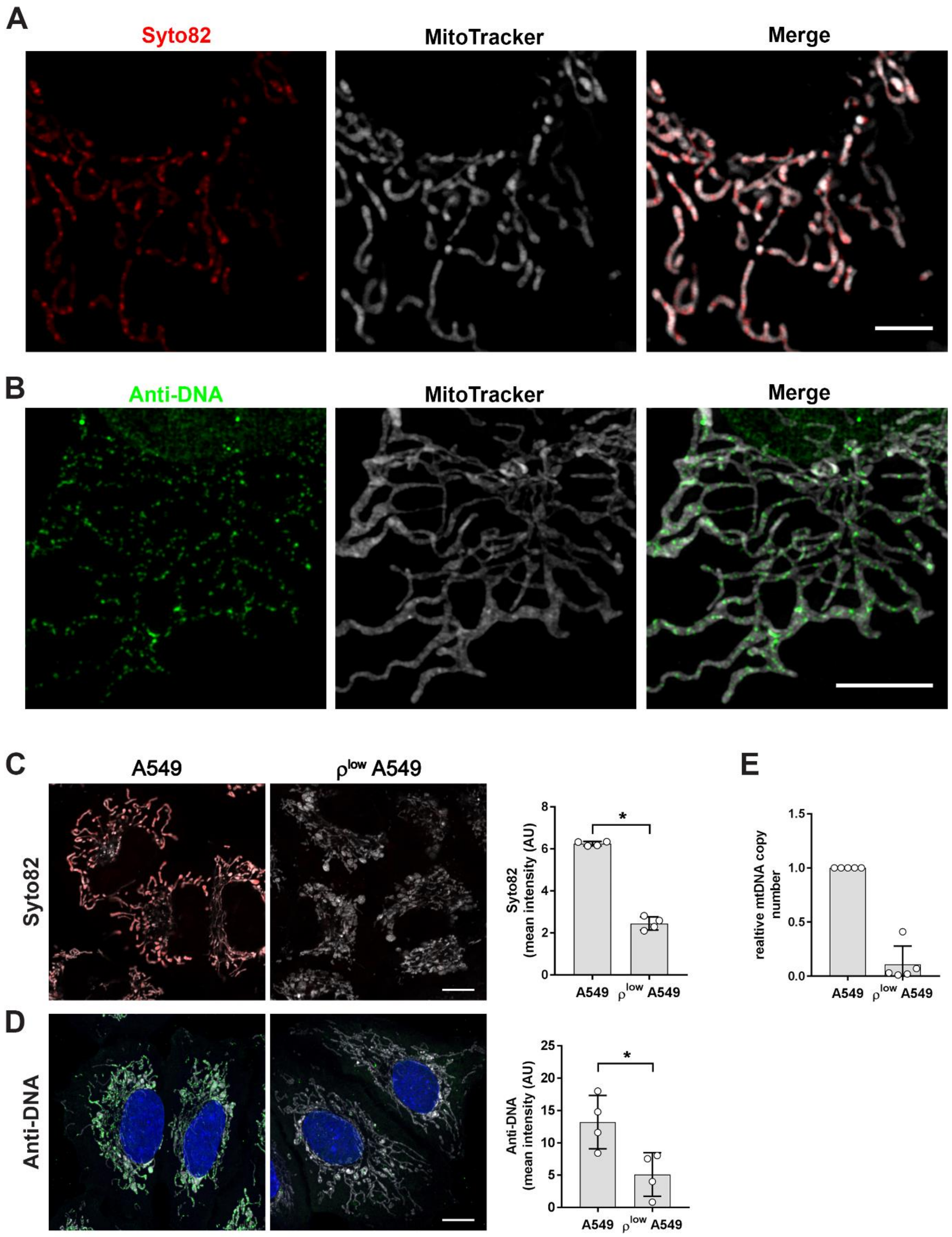

Supplementary Figure 3. Syto82 stains mitochondrial DNA in A549. (A) 2D image of a representative high-resolution microscopy (Airyscan) of mitochondria in living A549 stained with Syto82 (red) and MitoTrackerDeepRed (grey). The scale bar represents 5 µm. (B) Representative maximum intensity projection of a SIM 3D-stack

of mitochondria in A549 stained with anti-DNA antibody (green) and MitoTrackerOrange (grey). The scale bar represents 5 µm. (C) Representative deconvolved confocal 2D plane of living A549 and ρ^{low} A549 cells, respectively stained with Syto82 (red) and MitoTrackerDeepRed (grey). The scale bar represents 10 µm. Quantification of Syto82 fluorescence in A549 control cells and ρ^{low} A549 cells (mean ± SD from n = 4 independent experiments, *P < 0.05; one-tailed Mann-Whitney test). (D) Representative maximum intensity projection of a deconvolved confocal z-stack of A549 and ρ^{low} A549 cells, respectively stained with anti-DNA antibody (green) and MitoTracker Orange (grey). The nuclei were labelled with DAPI (blue). The scale bar represents 10 µm. Quantification of Anti-DNA fluorescence in A549 control cells and ρ^{low} A549 cells (mean ± SD from n = 4 independent experiments, *P < 0.05; one-tailed Mann-Whitney test). *P < 0.05; one-tailed Mann-Whitney test. (E) qPCR analysis of relative mtDNA copy number from control A549 and ρ^{low} A549 cells (mean ± SD from n = 5 independent experiments).

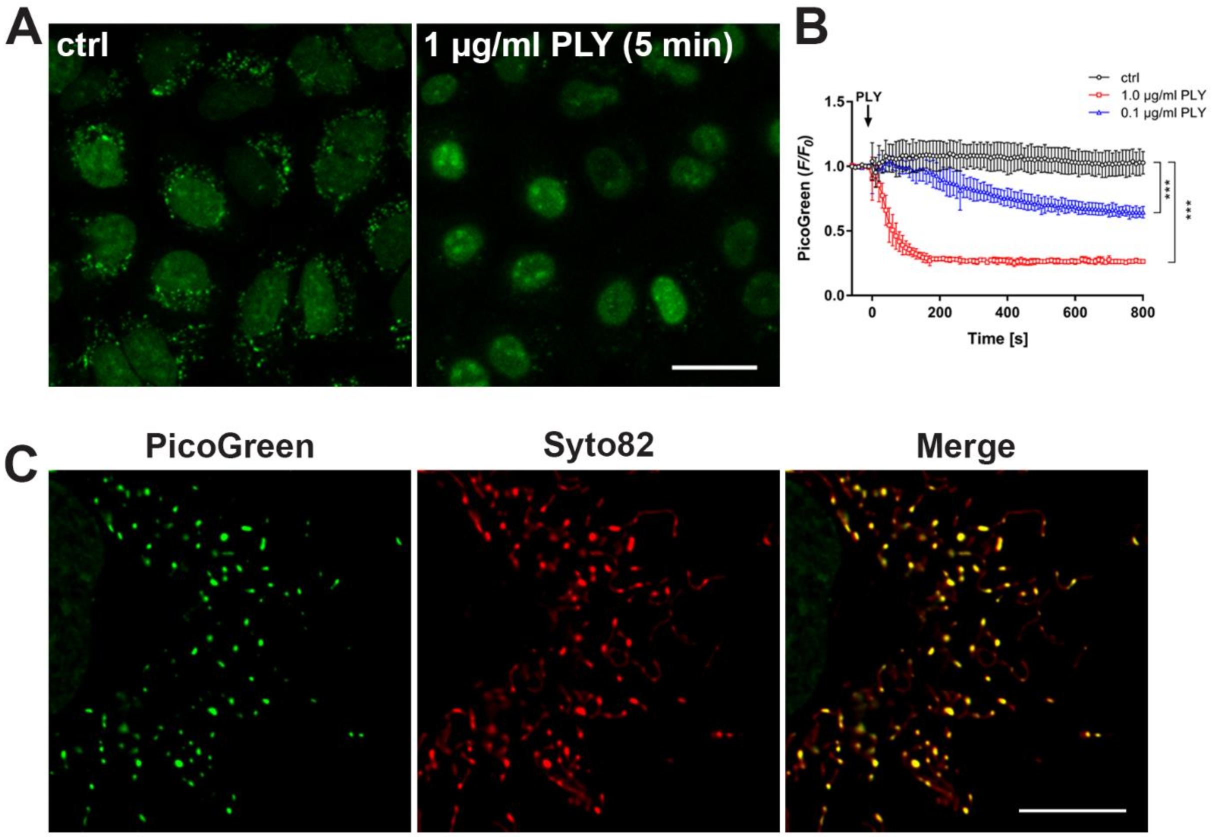

Supplementary Figure 4. Validation of PLY-induced mtDNA release using PicoGreen. (A) Representative confocal 2D plane of A549 cells stained with PicoGreen left unstimulated or stimulated with 1.0 µg/ml PLY for 5 min. The scale bar represents 25 µm. (B) Quantification of mtDNA release (PicoGreen F/F_0 ratios) in cells stimulated with 0.1 µg/ml and 1.0 µg/ml PLY, respectively or left unstimulated (mean ± SD from n = 4 independent experiments). (C) Representative 2D image acquired using spectral confocal microscopy in combination with linear unmixing of living A549 cells stained with PicoGreen (green) and Syto82 (red). The scale bar represents 10 µm.

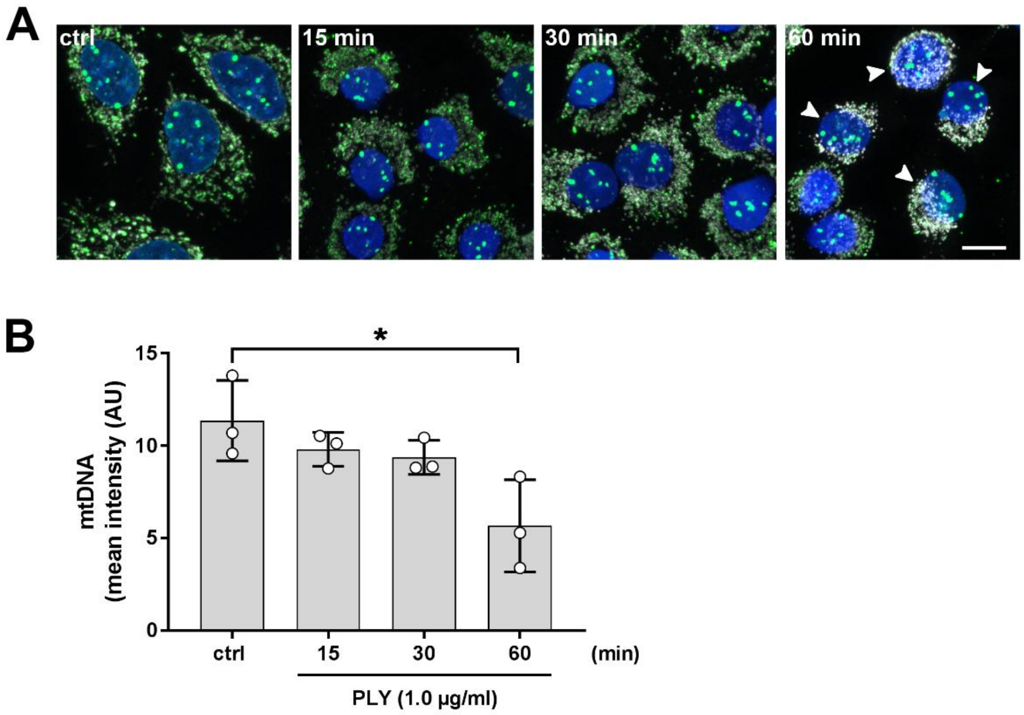

Supplementary Figure 5. Conventional immunofluorescence validates mtDNA release by PLY Conventional immunofluorescence. (A) Representative confocal images of A549 cells left unstimulated or stimulated with 1.0 µg/ml PLY for 15, 30, and 60 min. The nuclear DNA and nucleoids were stained with anti-DNA antibody (green), the mitochondrial network is stained with MitoTracker orange (grey). The nuclei were labelled with DAPI (blue). Arrowheads indicate cells with released DNA (loss of green signal) from mitochondria. Scale bar represents 10 µm. (B) Quantification of mtDNA signal within the mitochondrial network. Approximately 90 cells per condition were analysed in each experiment (n = 3 experiments, mean ± SD, *$P < 0.05$, Kruskal-Wallis test with Dunn's post-hoc test).

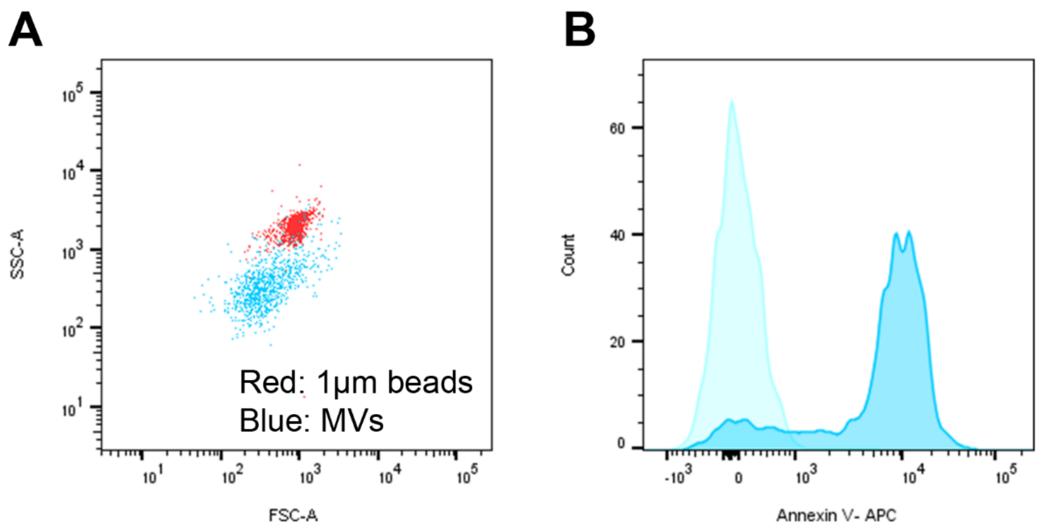

Supplementary Figure 6. Characterization of isolated microvesicles. (A) Representative MV population (blue events). To gate and define the size of the isolated MVs, 1 μm beads (red events) were used. (B) Representative MV population stained with annexin V (bright blue). In the presence of EDTA (negative control), MVs do not bind to annexin V (light blue). One representative experiment is shown.

Supplementary Movie Legends

Supplementary Movie 1. Mitochondrial morphology, caspase-3/7 activation, and PLY pores in pneumococcal A549 cell infection. A549 cells were infected with *S.pn.* D39Δ*cps* (moi 50, 6 h) and live-cell microscopy was performed using MitoTrackerOrange (yellow) and CellEvent™ Caspase-3/7 Green Detection Reagent (green). Cells were fixed afterwards and stained against PLY pores with specific antibodies (red). Scale bar, 5 µm.

Supplementary Movie 2. Mitochondrial morphology, caspase-3/7 activation, and PLY pores in PLY deficient infection of A549 cells. A549 cells were infected with *S.pn.* D39Δ*cps*Δ*ply* (moi 50, 6 h) and live-cell microscopy was performed using MitoTrackerOrange (yellow) and CellEvent™ Caspase-3/7 Green Detection Reagent (green). Cells were fixed afterwards and stained against PLY pores with specific antibodies (red). Scale bar, 5 µm.

Supplementary Movie 3. Mitochondrial morphology and motility in unstimulated human lung tissue. Time-lapse spectral confocal live-tissue imaging illustrating mitochondrial morphology and motility in normal alveolar epithelial cells of living human lung tissue. Mitochondria stained with MitoTrackerOrange are pseudocolored in cyan and autofluorescence is shown in grey. Scale bar, 5 µm.

Supplementary Movie 4. Mitochondrial morphology and motility in human lung tissue stimulated with PLY. Time-lapse spectral confocal live-tissue imaging illustrating mitochondrial motility in alveolar epithelial cells of living human lung tissue stimulated with 1 µg/ml PLY for 1 h. Mitochondria stained with MitoTrackerOrange are pseudocolored in cyan, caspase-3/7 activation is shown in magenta, and autofluorescence is shown grey. Scale bar, 5 µm.

Supplementary Movie 5. 3D volume rendering illustrating mitochondrial alterations and caspase-3/7 activation in human lung tissue infected with PLY-expressing pneumococci. Tissue was stimulated with PLY expressing *S.pn.* D39-GFP (10^6 cfu/ml) for 12 h hours (pseudocolored in red). Mitochondria, stained with MitoTrackerOrange are pseudocolored in cyan, caspase-3/7 is shown in magenta, and autofluorescence is shown in grey. In addition PLY was stained after fixation with antibodies in green. Scale bar, 5 µm.

Supplementary Movie 6. Influx of Calcium into mitochondria and activation of caspase 3/7 in A549 cells stimulated with 1.0 µg/ml PLY. A549 cells loaded with Rhod2-AM (red) and CellEvent™ Caspase-3/7 Green Detection Reagent (green) were stimulated with 1.0 µg/ml PLY and imaged by confocal time-lapse microscopy (DIC, grey). Images extracted at different time points are shown in Fig 3A. Scale bar, 25 µm.

Supplementary Movie 7. Influx of Calcium into mitochondria and activation of caspase 3/7 in A549 cells stimulated with 0.1 µg/ml PLY. A549 cells loaded with Rhod2-AM (red) and CellEvent™ Caspase-3/7 Green Detection Reagent (green) were stimulated with 0.1 µg/ml PLY and imaged by confocal time-lapse microscopy (DIC, grey). Scale bar, 25 µm.

Supplementary Movie 8. Mitochondrial calcium flux and activation of caspase 3/7 in unstimulated A549. A549 cells loaded with Rhod2-AM (red) and CellEvent™ Caspase-3/7 Green Detection Reagent (green) and imaged by confocal time-lapse microscopy (DIC, grey). Scale bar, 25 µm.

Supplementary Movie 9. $\Delta\Psi_m$ changes in A549 cells stimulated with 1.0 µg/ml PLY. A549 cells loaded with TMRE (red) were stimulated with 1.0 µg/ml PLY and imaged by confocal time-lapse microscopy (DIC, grey). Scale bar, 35 µm.

Supplementary Movie 10. $\Delta\Psi_m$ changes in A549 cells stimulated with 0.1 µg/ml PLY. A549 cells loaded with TMRE (red) were stimulated with 0.1 µg/ml PLY and imaged by confocal time-lapse microscopy (DIC, grey). Scale bar, 35 µm.

Supplementary Movie 11. $\Delta\Psi_m$ changes in unstimulated A549 cells. A549 cells loaded with TMRE (red) were imaged by confocal time-lapse microscopy (DIC, grey). Scale bar, 35 µm.

Supplementary Movie 12. Opening of the mPTP in A549 cells stimulated with 1.0 µg/ml PLY. A549 cells loaded with Calcein (green) and MitoTracker Orange (red) in the presence of $CoCl_2$ were stimulated with 1.0 µg/ml PLY and imaged by confocal time-lapse microscopy (DIC, grey). Scale bar, 35 µm.

Supplementary Movie 13. Opening of the mPTP in A549 cells stimulated with 0.1 µg/ml PLY. A549 cells loaded with Calcein (green) and MitoTracker Orange (red) in the presence of $CoCl_2$ were stimulated with 0.1 µg/ml PLY and imaged by confocal time-lapse microscopy (DIC, grey). Scale bar, 35 µm.

Supplementary Movie 14. Opening of the mPTP in unstimulated A549. A549 cells loaded with Calcein (green) and MitoTracker Orange (red) in the presence of $CoCl_2$ were imaged by confocal time-lapse microscopy (DIC, grey). Scale bar, 35 µm.

Supplementary Movie 15. Visualization of mtDNA in A549 cells stimulated with 1.0 µg/ml PLY. A549 cells loaded with Syto82 (red) and MitoTracker Green (pseudocolored grey) were stimulated with 1.0 µg/ml PLY and imaged by spectral confocal time-lapse microscopy. Scale bar, 35 µm.

Supplementary Movie 16. Visualization of mtDNA in unstimulated A549 cells. A549 cells loaded with Syto82 (red) and MitoTracker Green (pseudocolored grey) were imaged by spectral confocal time-lapse microscopy. Scale bar, 35 µm.

Supplementary References

1 Nikolaisen, J. *et al.* Automated quantification and integrative analysis of 2D and 3D mitochondrial shape and network properties. *PloS one* **9**, e101365, doi:10.1371/journal.pone.0101365 (2014).

2 Patrushev, M. *et al.* Mitochondrial permeability transition triggers the release of mtDNA fragments. *Cell Mol Life Sci* **61**, 3100-3103, doi:10.1007/s00018-004-4424-1 (2004).

3 Wlodkowic, D., Skommer, J. & Darzynkiewicz, Z. SYTO probes in the cytometry of tumor cell death. *Cytometry. Part A : the journal of the International Society for Analytical Cytology* **73**, 496-507, doi:10.1002/cyto.a.20535 (2008).

4 Iborra, F. J., Kimura, H. & Cook, P. R. The functional organization of mitochondrial genomes in human cells. *BMC biology* **2**, 9, doi:10.1186/1741-7007-2-9 (2004).

5 King, M. P. & Attardi, G. Human cells lacking mtDNA: repopulation with exogenous mitochondria by complementation. *Science (New York, N.Y.)* **246**, 500-503 (1989).

6 Coppey-Moisan, M., Brunet, A. C., Morais, R. & Coppey, J. Dynamical change of mitochondrial DNA induced in the living cell by perturbing the electrochemical gradient. *Biophysical journal* **71**, 2319-2328, doi:10.1016/s0006-3495(96)79472-9 (1996).

7 Ashley, N., Harris, D. & Poulton, J. Detection of mitochondrial DNA depletion in living human cells using PicoGreen staining. *Experimental cell research* **303**, 432-446, doi:10.1016/j.yexcr.2004.10.013 (2005).

8 Garrido, N. *et al.* Composition and dynamics of human mitochondrial nucleoids. *Molecular biology of the cell* **14**, 1583-1596, doi:10.1091/mbc.E02-07-0399 (2003).

9 van de Corput, M. P. *et al.* Detection of mitochondrial DNA deletions in human skin fibroblasts of patients with Pearson's syndrome by two-color fluorescence in situ hybridization. *The journal of histochemistry and cytochemistry : official journal of the Histochemistry Society* **45**, 55-61, doi:10.1177/002215549704500108 (1997).

10 Legros, F., Malka, F., Frachon, P., Lombes, A. & Rojo, M. Organization and dynamics of human mitochondrial DNA. *Journal of cell science* **117**, 2653-2662, doi:10.1242/jcs.01134 (2004).

11 Hayashi, J., Takemitsu, M., Goto, Y. & Nonaka, I. Human mitochondria and mitochondrial genome function as a single dynamic cellular unit. *The Journal of cell biology* **125**, 43-50 (1994).
